# Cdc7 kinase stimulates Aurora B kinase in M-phase

**DOI:** 10.1038/s41598-019-54738-2

**Published:** 2019-12-09

**Authors:** Sayuri Ito, Hidemasa Goto, Kinue Kuniyasu, Mayumi Shindo, Masayuki Yamada, Kozo Tanaka, Gaik-Theng Toh, Masaaki Sawa, Masaki Inagaki, Jiri Bartek, Hisao Masai

**Affiliations:** 1 Department of Genome Medicine, Tokyo Metropolitan Institute of Medical Science, Tokyo, 156-8506 Japan; 2 Department of Neural Regeneration and Cell Communication, Mie University Graduate School of Medicine, Tsu, Mie 514-8507 Japan; 3 Department of Molecular Oncology, Institute of Development, Aging and Cancer, Tohoku University, Sendai, 980-8575 Japan; 4 Laboratory of Protein Analyses, Tokyo Metropolitan Institute of Medical Science, Tokyo, 156-8506 Japan; 5 Medical Education Center, Graduate School of Medicine, Kyoto University, Kyoto, Japan; 6 Institute of Molecular and Translational Medicine, Faculty of Medicine and Dentistry, Palacky University, 77900 Olomouc, Czech Republic; 7 Carna Biosciences, Inc., Kobe, Japan; 8 Department of Physiology, Mie University Graduate School of Medicine, Tsu, Mie 514-8507 Japan; 9 Danish Cancer Society Research Center, Copenhagen, Denmark; 10 Division of Genome Biology, Department of Medical Biochemistry and Biophysics, Karolinska Institute, Stockholm, Sweden

**Keywords:** Chromosome segregation, Chromosome segregation

## Abstract

The conserved serine-threonine kinase, Cdc7, plays a crucial role in initiation of DNA replication by facilitating the assembly of an initiation complex. Cdc7 is expressed at a high level and exhibits significant kinase activity not only during S-phase but also during G2/M-phases. A conserved mitotic kinase, Aurora B, is activated during M-phase by association with INCENP, forming the chromosome passenger complex with Borealin and Survivin. We show that Cdc7 phosphorylates and stimulates Aurora B kinase activity *in vitro*. We identified threonine-236 as a critical phosphorylation site on Aurora B that could be a target of Cdc7 or could be an autophosphorylation site stimulated by Cdc7-mediated phosphorylation elsewhere. We found that threonines at both 232 (that has been identified as an autophosphorylation site) and 236 are essential for the kinase activity of Aurora B. Cdc7 down regulation or inhibition reduced Aurora B activity *in vivo* and led to retarded M-phase progression. SAC imposed by paclitaxel was dramatically reversed by Cdc7 inhibition, similar to the effect of Aurora B inhibition under the similar situation. Our data show that Cdc7 contributes to M-phase progression and to spindle assembly checkpoint most likely through Aurora B activation.

## Introduction

The conserved serine-threonine kinase, Cdc7-Dbf4, phosphorylates Mcm in the pre-Replicative Complex (pre-RC) and facilitates recruitment of other replication factors including Cdc45 for initiation of DNA replication^1–8^. Cdc7 forms a complex with an activation subunit, Dbf4/ASK, to become an active kinase^9–11^. Cdc7 also plays a role in activation of replication checkpoint pathway (ATR-Claspin-Chk1 pathway in vertebrates^12–16^). Recent studies show that Cdc7 kinase plays roles in various chromosome transactions including meiotic recombination, meiosis, trans-lesion DNA synthesis, and histone modification^17,18^. Cdc7 also interacts with various chromosome markers including HP1 that recognizes methylated lysine 9 of histone H3 and with centromere proteins^19,20^. Cdc7 also phosphorylates Histone H3 at threonine-45 as a part of replication stress responses^21^.

Aurora B, a conserved mitotic kinase, is activated during M-phase by association with INCENP^22^, forming the chromosome passenger complex (CPC) with Borealin and Survivin^23–28^. It plays critical roles in chromosome separation and cytokinesis by translocating from chromatin to centromere to mid-body. During prophase, Aurora B is spread on chromatin through INCENP bound to HP1. During prometaphase, CPC, phosphorylated by Cdk1, binds to Shugosin, and localizes at inner centromere^29^. This step requires Aurora B kinase, as well as Bub1 and Haspin kinases. Aurora B is also required for maintenance of Spindle Assembly Checkpoint (SAC) and cytokinesis.

Other mitotic kinase, Plk1, also plays an important role in mitotic events including mitotic entry, centrosome maturation, bipolar spindle formation, correct microtubule attachment to kinetochore, and cytokinesis^30,31^. Plk1 is recruited to the kinetochore through INCENP phosphorylated by Cdk1 and collaborates with Aurora B^32^. Plk1 phosphorylates Survivin at Ser 20 for further Aurora B kinase activation in centromere^33^. Cdc7 is expressed at a high level and exhibits significant kinase activity during G2/M-phases^34,35^, although its role, if any, in the mitotic phase has not been known.

Here, we show that Cdc7 phosphorylates Aurora B and stimulates its kinase activity. We also show that Aurora B activation in early M-phase by Cdc7 contributes to efficient M-phase progression and to efficient operation of SAC.

## Results

### Cdc7-ASK phosphorylates aurora B and stimulates its kinase activity *in vitro*

**Figure 1:**
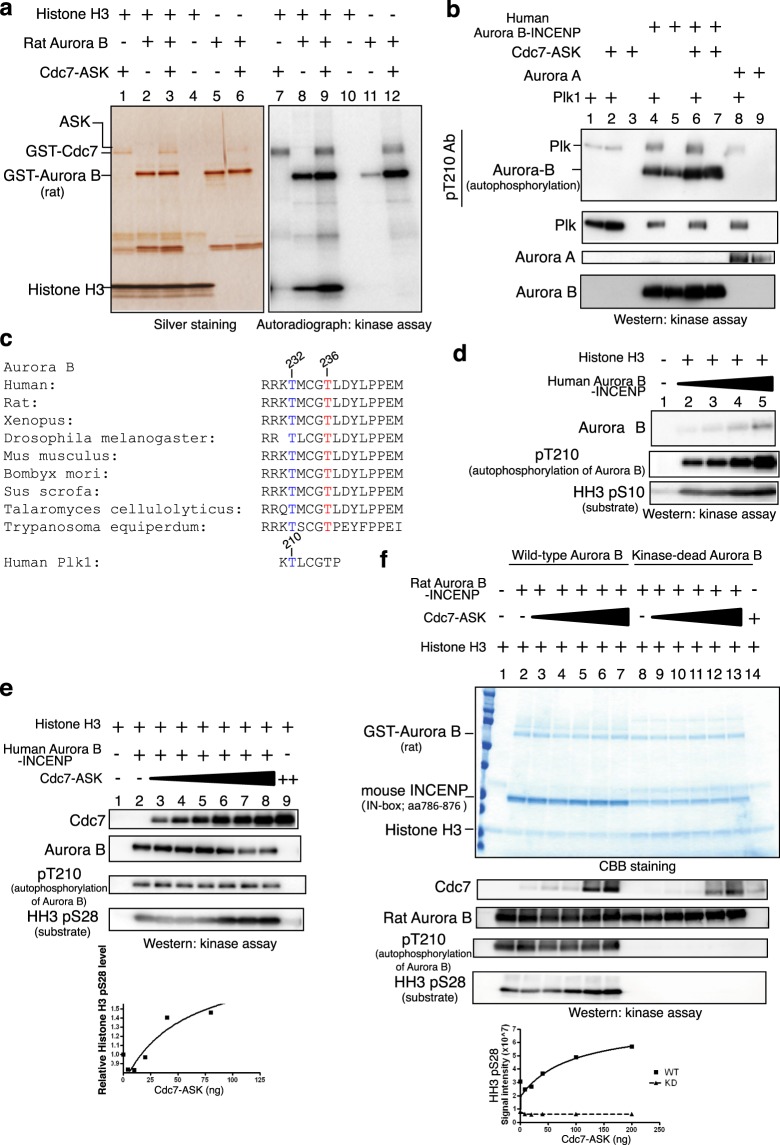
Cdc7-ASK phosphorylates Aurora B and increases its kinase activity *in vitro*. (**a**) Rat Aurora B (50 ng), Cdc7-ASK (20 ng) and Histone H3 (HH3; 0.5 µg as a substrate) were mixed as indicated in the kinase assays with [γ-^32^P] ATP. Half of the reaction was analyzed. HH3 was phosphorylated by Aurora B kinase. Cdc7-ASK increases phosphorylation of both HH3 and Aurora B. (**b**) Human Aurora B (50 ng), Cdc7-ASK (20 ng), and Plk1 (50 ng) were mixed as indicated in kinase assays. Phosphorylation was detected by western analyses using pT210 (detecting the autophosphorylation of Aurora B; see **c**). Proteins were also detected by western. (**c**) Comparison of the amino acid sequences around the T-loop of Aurora B from different species. Thr232 and Thr236 of human Aurora B are conserved. Thr232 is the known autophosphorylation site^22,36^. The sequences around Thr232 are similar to those around T210 of Plk. Thus, pT210 antibody detects Aurora B T232 phosphorylation. (**d**) Titration of human Aurora B-INCENP in the kinase assays with HH3 as a substrate. Phosphorylation was detected by pT210 and HH3 pS10 antibodies. (**e**) Titration of Cdc7-ASK in the kinase assays with human Aurora B-INCENP complex. Cdc7 increases HH3 S28 phosphorylation, which was detected by HH3 pS28 antibody. (**f**) Rat Aurora B (wild-type or kinase-dead [K109R]) in a complex with INCENP purified from insect cells was incubated with Cdc7-ASK. Cdc7 increased HH3 phosphorylation detected by pS28 antibody. The pT210 signal in the wild-type Aurora B was not significantly affected by Cdc7 in this experiment. pT210 and HH3 pS28 were undetectable with the kinase-dead Aurora B. In (**b**,**d**,**e)**, human Aurora B-INCENP complex from Carna was used, and in (**a**,**b**,**e**,**f**), Cdc7-ASK complex from Carna was used.

We conducted *in vitro* Cdc7 kinase assays using purified rat Aurora B or human Aurora B/INCENP complex as a substrate. The kinase activity of the rat-Aurora B, as measured by phosphorylation of Histone H3 (HH3), significantly increased in the presence of human Cdc7-ASK (Fig. 1a, lanes 8 and 9). Phosphorylation of Aurora B increased in the presence of Cdc7 (Fig. 1a, lanes 11 and 12), and this may be due to Cdc7-mediated direct phosphorylation and/or to increased autophosphorylation activity of Aurora B. In an assay using a peptide substrate (Kemptide), two different preparations of Cdc7-ASK stimulated the phosphorylation of this peptide by 1.5 fold (Supplementary Fig. S1a). On the other hand, the kinase activity of Plk1, measured in a similar assay, was not affected by Cdc7-ASK *in vitro* (Supplementary Fig. S1b). Anti-Plk1 (phospho-Thr210) antibody, raised against the phosphorylated Thr210 of human Plk1 (Fig. 1b), can react with phosphorylated Aurora B likely due to the presence of the similar amino acid stretch around Thr232 (Fig. 1c). Indeed, the auto phosphorylated Aurora B could be detected by this antibody (Fig. 1b,d). Cdc7 increased *in vitro* phosphorylation of Histone H3 S28 by the human Aurora B/INCENP, but did not affect or only slightly increased the autophosphorylation level of Aurora B detected by anti-Plk1-pT210 antibody (Fig. 1b,e). Similar results were obtained using rat Aurora B-INCENP complex purified from insect cells (Fig. 1f).

The phosphorylation sites of human and rat Aurora B by Cdc7 kinase were determined by mass spectrometry analysis (Supplementary spreadsheet). Phosphorylation of Thr236 was detected in human Aurora B phosphorylated by Cdc7. The Thr235 of rat Aurora B (corresponding to Thr236 in human) was also phosphorylated by Cdc7. This threonine is located very close to the auto-phosphorylation site, T232, in human Aurora B. The amino acids surrounding these threonines are very conserved among species (Fig. 1c). The results suggest that T236 may be a critical phosphorylation site targeted by Cdc7. However, at present we cannot rule out the possibility that T236 is an autophosphorylation site stimulated by Cdc7-mediated phosphorylation (see Discussion).

### Mutation at T232 or T236 results in loss of Aurora B kinase activity

**Figure 2:**
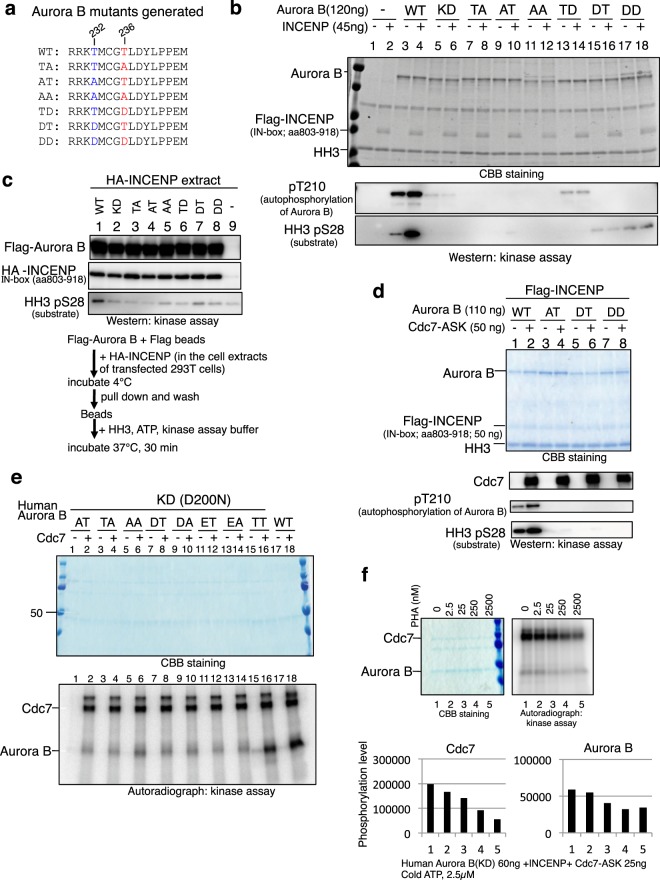
Both T232 and T236 of human Aurora B are important for its kinase activity *in vitro*. (**a**) Mutations introduced at T232 and T236 in human Aurora B. The mutants of the Aurora B were expressed as C-terminally Flag-tagged forms^72^ in 293 T cells and purified by using Flag-antibody beads. (**b**) Flag-tagged human Aurora B, as indicated, and Flag-tagged INCENP (IN-box polypeptide; aa803-918) proteins were separately purified from 293 T cells. The purified Aurora B was incubated with HH3 in the presence or absence of INCENP. WT, wild-type; KD, kinase-dead (D200N; D200 of the conserved HRD was changed to N). (**c**) The 293 T extracts containing HA-tagged INCENP (IN-box polypeptide) and purified Flag-tagged Aurora B mutants, as indicated, were mixed and immunoprecipitated with anti-Flag antibody, followed by kinase assays in the presence of HH3 and ATP. The presence of HA-INCENP in the immunoprecipitates was confirmed by western blotting using anti-HA antibody. Phosphorylation was detected by HH3 pS28 antibody. (**d**) The wild-type and mutant Aurora B proteins, as indicated, and INCENP were incubated in the presence or absence of Cdc7-ASK. In (**b**,**c**,**d)**, phosphorylations were detected by western analyses using pT210 and HH3 pS28 antibodies. (**e**) Human Aurora B KD (D200N) was further mutated at Thr232 and Thr236. These human Aurora B (60 ng) mutants were incubated in *in vitro* kinase assays with [γ-^32^P] ATP in the absence or presence of Cdc7-ASK (25 ng). (**f**) Human Aurora B KD (60 ng), INCENP and Cdc7/ASK (25 ng) were incubated in kinase assays with [γ-^32^P] ATP. Increasing concentrations of a Cdc7 inhibitor (PHA-767491) were added, as shown. A long exposure of the autorad panel is shown in Supplementary Fig. S8.

We have substituted the two threonines (T232 and T236) with either alanine (A) or aspartic acid (D) in various combinations (Fig. 2a). Alanine substitutions would generate non-phosphorylatable mutants, whereas the aspartic acid substitution is expected to generate mutants mimicking the phosphorylated state (phospho-mimic mutants).

Aurora B-mediated phosphorylation of HH3 was significantly stimulated by the presence of INCENP (IN-box polypeptide), consistent with previous reports^36,37^ (Supplementary Fig. S2a). The kinase-dead Aurora B did not exhibit phosphorylation activity toward HH3 even in the presence of INCENP, as expected (Fig. 2b, lanes 5 and 6; however, it should be noted that there is remaining autophosphorylation activity in this KD mutant [D200N]; see also lane 15 of Fig. 2e). Judged by Aurora B-T232 (detected by anti-Plk1-pT210 antibody) and HH3-Ser28 phosphorylation, the catalytic activity of Aurora B was stimulated by the addition of INCENP-IN-box, but was inhibited by excess INCENP (Supplementary Fig. S2a, lanes 11 and 12). The optimal Aurora B:INCENP ratio was 1:1 in this assay.

We conducted kinase assays with above mutants in the absence and presence of INCENP polypeptide. In contrast to the wild-type Aurora B, very little activity was observed with the mutants except for the weak Aurora B-pT232 signal on TD and weak HH3 pS28 signals with DT and DD. Autophosphorylation of TD and HH3 phosphorylation by DT was not stimulated by the presence of INCENP (Fig. 2b, lanes 13–16), and HH3 phosphorylation by DD was slightly stimulated by INCENP (Fig. 2b, lanes 17 and 18). These results suggest that both 232 and 236 threonines are important for Aurora B kinase activity. The substitutions with aspartic acid did not mimic the phosphorylated state, but instead resulted in attenuated kinases. To exclude the possibility that the Aurora B mutants do not show kinase activity due to their inability to bind to INCENP, we tested the interaction between the purified Flag-tagged Aurora B and HA-INCENP (in the cell extracts). Immunoprecipitation by the Flag antibody indicates that all the mutants interact with INCENP with similar affinity (Fig. 2c), showing that reduced kinase activities of the mutants are due to intrinsic deficiency of the catalytic subunit. The T232/T236 mutants exhibited very much reduced phosphorylation of the substrate protein in kinase assays using radioactive ATP as well (data not shown). Cdc7 stimulated the kinase activity of the wild-type Aurora B in the presence of purified INCENP, but not that of AT, DT or DD mutant (Fig. 2d). Although Aurora B KD does not have its kinase activity, Cdc7 can phosphorylate Aurora B KD (Fig. 2e lane16), if both 232 and 236 are threonines. Cdc7 can also phosphorylate both WT and KD Aurora B purified from *E. coli* (data not shown). Incubation of human KD Aurora B, INCENP and Cdc7 with Cdc7 inhibitor (PHA-767491) resulted in reduction of both Cdc7 and Aurora B phosphorylation levels in a dose dependent manner (Fig. 2f). The Cdc7 inhibitor does not affect the autophosphorylation of Aurora B and Aurora B-mediated HH3 phosphorylation (data not shown). These results confirm that the presence of both threonines is required for efficient Cdc7-mediated phosphorylation and activation of Aurora B.

### Cdc7 stimulates Aurora B kinase activity in M-phase cells

**Figure 3:**
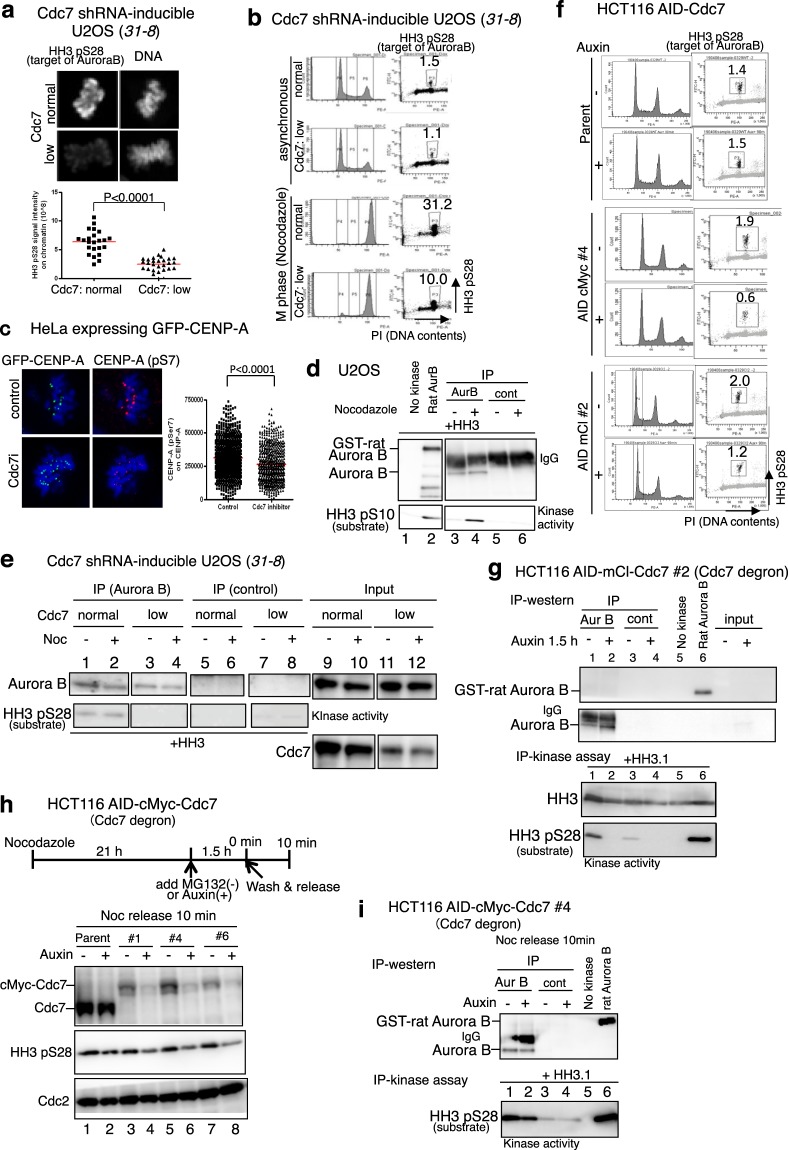
Cdc7 inhibition reduces HH3 pS28 and CENP-A pS7 in M-phase cells. (**a,b**,**e**) *31-8*, U2OS derivative carrying shRNA under an inducible promoter, was incubated with or without doxycycline for three days to reduce Cdc7 level. “low” represents Cdc7-depleted cells (with doxycycline), whereas “normal” represents control cells (without doxycycline). (**a**) HH3 pS28 signal and Hoechst33342 (DNA) during M-phase are shown (upper panels). HH3 pS28 signal on chromatin were calculated by Imaris software and plotted by the Prism software (lower graph). (**b**) *31-8* cells (normal and low) were either treated with 100 ng/ml nocodazole or DMSO for 20 hrs before analyses by FACS. Western analyses of protein expression are shown in Supplementary Fig. S3a,b. (**c**) HeLa cells expressing GFP-CENP-A were treated with 10 µM Cdc7 inhibitor (PHA-767491) for 100 min, fixed and stained with anti CENP-A (pSer7) antibody. Cells were observed under LSM780 confocal microscopy. Imaris Spot analyses were performed to determine the Alexa 546 signal (pSer7) intensity on GFP signal (CENP-A) spots. Control, n = 784; Cdc7 inhibitor treated, n = 561 (**d**) U2OS cells were incubated with 50 ng/ml nocodazole or DMSO (control) for 15 hrs. The IP-kinase assays were conducted using HH3 as a substrate. (**e**) *31-8* cells (normal and low) were non-treated (−) or treated with 30 ng/ml nocodazole for 45 min (+). The IP-kinase assays were conducted using HH3 as a substrate. (**f**) Parent and AID-Cdc7 HCT116 cells, treated with or without Auxin for 60 min (cMyc#4) or 90 min (mCl#2 and Parent), were analyzed by FACS. (**g**) AID-Cdc7 HCT116 (mCl#2) cells were treated with Auxin for 1.5 hrs. The IP-kinase assays were performed using HH3.1 as a substrate. Western blot analyses of the extracts used are shown in Supplementary Fig. S3f. A long exposure of the Aurora B blot is shown in Supplementary Fig. S8. (**h**) AID-cMyc-Cdc7 (#1, #4 and #6) and parent HCT116 cells arrested with nocodazole were treated with MG132 (−) or Auxin (+) for 1.5 hrs. Western blotting was performed as indicated. (**i**) The CSK-soluble extracts of #4 clone prepared in (**h**) were used for IP-kinase assay. Purified GST-Aurora B (rat) was used as a control for the kinase assays (lane 6).

U2OS (*31-8*) cell line was established in which Cdc7 shRNA can be inducibly expressed by addition of doxycycline (Supplementary Fig. S3a). In this cell line, the HH3 pS28 signal, which is generated by Aurora B on M-phase chromatin, was decreased by addition of doxycycline (Cdc7 “low’ in Fig. 3a). Nocodazole-treated M-phase cells showed increased HH3 pS28 as shown by FACS analyses (Fig. 3b). The intensities of HH3 pS28 signals during M-phase were also reduced by Cdc7 down regulation in *31-8* cells (Fig. 3b, Supplementary Fig. S3b,c). Although Cdc7 down regulation in *31-8* reduced HH3 pS28, INCENP signal localization or the intensity on chromatin did not change (Supplementary Fig. S3d). Furthermore, Aurora B pT232 auto-phosphorylation signal intensity on chromatin was not affected by Cdc7 down regulation in *31-8* or by Cdc7 siRNA treatment (Supplementary Fig. S3d,e), consistent with the *in vitro* results (Fig. 1e,f). We also examined the effect of Cdc7 on CENP-A Ser7 phosphorylation, which is also phosphorylated by Aurora B, in M-phase cells. CENP-A pS7 levels in GFP-CENP-A expressing HeLa cells were reduced by Cdc7 inhibitor treatment (Fig. 3c).

Aurora B was immunoprecipitated in nocodazole-treated or control U2OS cells with a specific antibody and kinase assays were performed with HH3 as a substrate. Nocodazole-treated M-phase cells showed higher Aurora B activity than control cells (Fig. 3d, compare lanes 3 and 4). We then conducted similar kinase assays in U20S (*31-8*) in which Cdc7 can be downregulated by inducing shRNA expression. The HH3 phosphorylation was reduced by Cdc7 depletion (Fig. 3e, compare lanes 1 and 3, lanes 2 and 4), consistent with the notion that Cdc7 regulates the phosphorylation activity of Aurora B kinase in cells.

To exclude the S phase effect of Cdc7, we constructed AID-tagged Cdc7 cell lines, in which Cdc7 protein level can be acutely reduced by addition of Auxin (Supplementary Fig. S3f). HH3 pS28 levels in M-phase were also reduced in Auxin treated AID-tagged Cdc7 HCT116 cells (Fig. 3f). In these cell lines Cdc7 degradation occurred rapidly after Auxin addition (Fig. 3g–i, Supplementary Figs. S3f and S4d).

Using one of the AID-tagged Cdc7 cell lines, we showed that Cdc7 depletion by Auxin reduced the Aurora B kinase activity in 1.5 hrs (Fig. 3g, compare lanes 1 and 2). In three independent HCT116 AID-cMyc-Cdc7 cells released for 10 min from nocodazole-induced M-phase arrest, the cellular HH3 pS28 level was reduced in Auxin-treated cells compared to control cells (Fig. 3h, lanes 4,6,8 compared to lanes 3,5,7, respectively). In the same cells, Aurora B kinase activity, measured by IP-kinase assay, was also reduced in the Auxin-treated sample (Fig. 3i, compare lanes 1 and 2). All these results support the conclusion that Cdc7 is required for efficient Aurora B kinase activity during M-phase.

**Figure 4:**
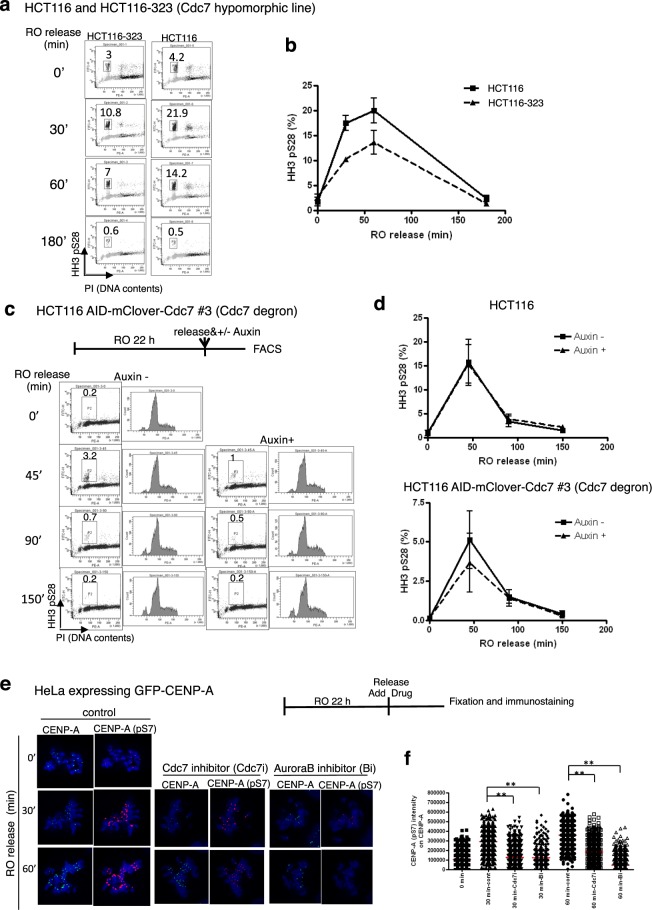
Cdc7 is required for Aurora B activation during M-phase. (**a,b**) The cells from HCT116 or HCT116-323 (Cdc7 promoter mutant with reduced Cdc7 protein level) were treated with RO-3306 overnight, released and collected at indicated time. The HH3 pS28 levels were analyze by FACS (**a**) and the averages of three independent experiments (measured by Prism software) are shown (**b**). Western blotting is shown in Supplementary Fig. S4a. (**c,d**) The cells from AID-tagged mClover Cdc7 clone #3 or parent HCT116 were arrested with RO-3306 overnight, washed three times, incubated in medium with/without Auxin and harvested at indicated time. The HH3 pS28 levels were analyzed by FACS (**c**) and the averages of three independent experiments (measured by Prism software) are shown (**d**). Cdc7 protein levels were analyzed by Western (Supplementary Fig. S4d). (**e,f**) HeLa cells expressing GFP-CENP-A were cultured in glass chamber slide, arrested with RO-3306, washed and released in the presence of various drugs. Control, no drug; Cdc7 inhibitor, 10 µM PHA-767491; Aurora B inhibitor, 100 nM AZD1152. Cells were fixed and immunostained by anti-CENP-A pS7 antibody (red) and Hoechst33342 (blue) and signals were detected by LSM780 confocal microscopy (**e**). Imaris spot analyses were conducted (**f**). 0 min, n = 732; 30 min-cont, n = 665; 30 min-Cdc7i, n = 1049; 30 min-Bi, n = 322; 60 min-cont, n = 680; 60 min-Cdc7i, n = 837; 60 min-Bi, n = 648; **p < 0.0001.

Next, we examined the effect of Cdc7 down regulation after release from RO-3306 arrest^38^. For that, we have generated a derivative of HCT116, HCT116-323 in which Cdc7 protein level is reduced due to a promoter mutation generated by CRISPR-Cas9 (to be described elsewhere). Thus, HCT116-323 is a Cdc7 hypomorphic derivative cell line. HCT116-323 was treated with a CDK inhibitor, RO-3306, and released into mitosis. The level of HH3 S28 phosphorylation was reduced in HCT116-323 compared to the parent HCT116 (Fig. 4a,b, Supplementary Fig. S4a,b). AID-tagged Cdc7 and parent cells were also treated with RO-3306, released into mitosis in the presence or absence of Auxin. HH3 pS28 was reduced in Auxin-treated cells (Figs. 4c,d lower panel, Supplementary Fig. S4d). In the parent HCT116 cells, the HH3 pS28 levels did not change by addition of Auxin (Fig. 4d, upper panel). CENP-A Ser7 phosphorylation, another Aurora B-mediated phosphorylation event, was also reduced in Cdc7 inhibitor-treated cells after release from RO-3306 arrest (Fig. 4e,f). These results also support the idea that Cdc7 is required for maximum Aurora B kinase activity during M-phase.

### The function of Cdc7 kinase is important for early M-phase progression

**Figure 5:**
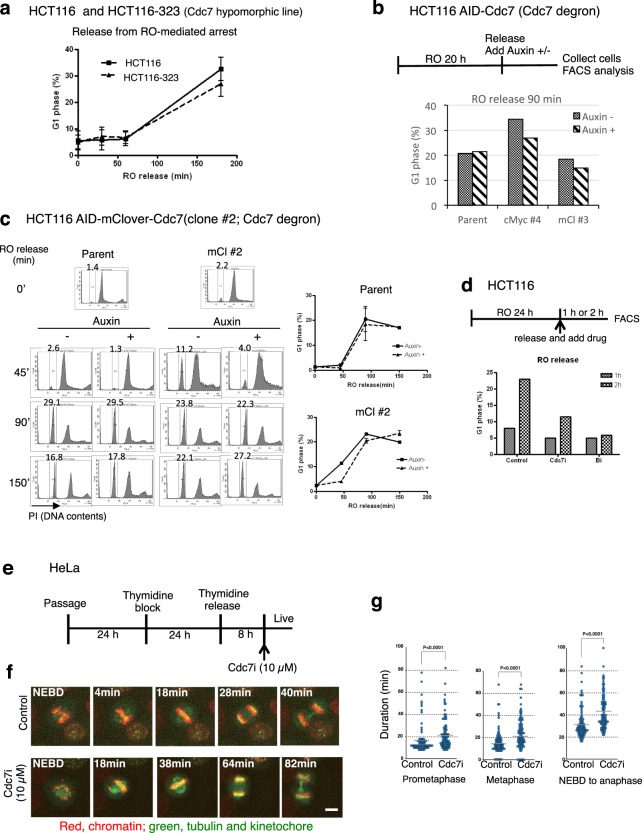
Cdc7 is required for efficient M-phase progression. (**a**) HCT116 and HCT116-323 were grown as described in Fig. 4a. The fractions of G1 phase populations were quantified from FACS analyses, and are shown at each timepoint after release from RO-3306 arrest. (**b**) AID-tagged-Cdc7 and parent HCT116 cells were grown as described in Fig. 4c. The fractions of G1 phase population at 90 min after release from RO-3306 arrest are shown. FACS data are shown in Supplementary Fig. S4e. (**c**) AID-mClover-Cdc7 #2 clone and parent HCT116 cells were grown as described in Fig. 4c. The fractions of G1 phase population are indicated in each panel and are plotted in the graphs. (**d**) HCT116 cells were released from RO-3306 arrest and cultured in fresh medium containing drugs indicated for one or two hrs, harvested and analyzed by FACS. The fractions of G1 phase populations are shown. Control, DMSO; Cdc7i, 10 µM PHA-767491; Bi, 100 nM of AZD1152. (**e–g**) HeLa cells expressing fluorescent protein labeled-kinetochore, -chromatin and -tubulin were synchronized with thymidine, released for 8 hrs and then a Cdc7 inhibitor (PHA-767491) was added at 10 µM. Live cell images were recorded for both control and drug-treated cells (Supplementary Movies 1 and 2, Supplementary Fig. S5). Red, chromatin; green, tubulin and kinetochore. The scheme of the experiment (**e**) and the pictures of a representative cell undergoing the nuclear envelope break down (NEBD) to cell division with times after NEBD shown in each panel (**f**). Durations of prometaphase, metaphase and NEBD to anaphase are shown for both control and Cdc7 inhibitor-treated cells (**g**). About one hundred mitotic cells were observed for each condition (Supplementary Fig. S5a**)**. The median is indicated with a bar. *P* values were obtained using Mann-Whitney *U* test.

HCT116-323 hypomorphic cells or Cdc7-depleted AID-tagged Cdc7 cells showed delayed G1 phase entry in release from RO-3306 arrest (shown by FACS analysis; Fig. 5a–c, Supplementary Fig. S4a,b,e). Treatment with Cdc7 inhibitor (PHA-767491) in release from RO-3306 arrest also delayed G1 phase entry (Fig. 5d). On the other hand, there was no significant effect of Cdc7 reduction on M-phase progression in cells released from nocodazole arrest (Supplementary Fig. S4a,c). These results suggest that Cdc7 is important for early M-phase progression.

In order to examine the effect of Cdc7 depletion on M-phase progression, HeLa cells expressing fluorescent protein labeled-kinetochore, -chromatin and -tubulin were released from thymidine block for 8 hrs and time-laps analyses were conducted with or without a Cdc7 inhibitor (PHA-767491). Quantification of the time-laps images indicated that both prometa- and meta-phase progression were prolonged by the presence of the Cdc7 inhibitor (Fig. 5e–g, Supplementary Fig. S5a, Supplementary Movies 1 and 2). Abnormal chromosome separation events (lagging chromosome and chromosome bridge) were also counted from the time-laps images. The numbers of abnormal chromosome separation slightly increased in the presence of the inhibitor; out of 103 cells, 14 or 5 cells showed lagging chromosomes or chromosome bridges, respectively, in control cells, whereas 17 or 7 cells, respectively, did in the cells treated with the Cdc7 inhibitor (PHA-767491). However, the difference was not statistically significant. Furthermore, we studied the effect of Cdc7 inhibition on error correction of kinetochore-microtubule attachment, and did not observe any significant differences from the control (Supplementary Fig. S5b, Supplementary Movies 3 and 4).

### Cdc7 regulates spindle assembly checkpoint through Aurora B

**Figure 6:**
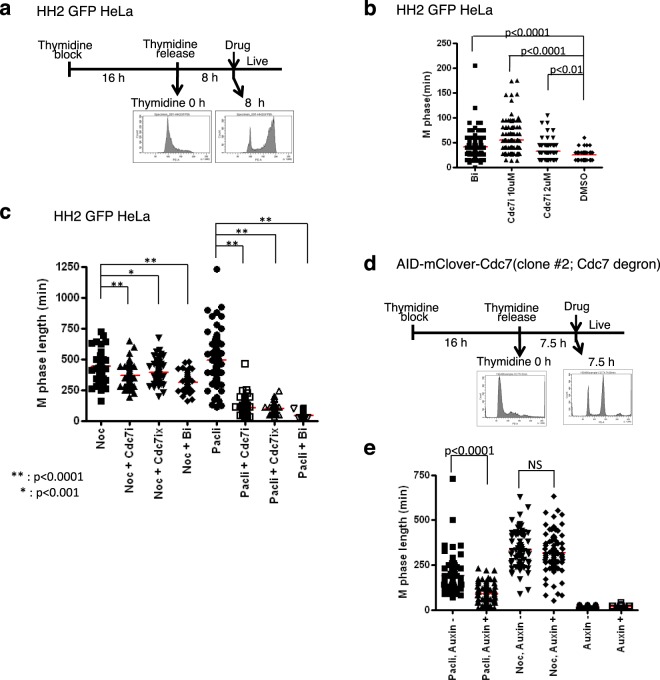
Cdc7 activity is required for spindle assembly checkpoint. Time-laps images were taken by Olympus LCV110 time-laps microscopy^74^. M-phase duration was determined for each cell by measuring the time from formation of round cells with condensed chromosomes to separation of daughter cells (cytokinesis) or decondensation of the chromosomes. (**a–c**) HeLa cells expressing HH2 GFP. Scheme of the experiment along with representative FACS profiles at the end of thymidine block and at 8 hrs after release (**a**). Movies are shown in Supplementary Movies 5–12. Representative images from the movie are shown in Supplementary Fig. S6a,b. (**d**,**e**) AID-mClover-Cdc7 clone #2. The scheme of the experiment along with representative FACS profiles at the end of thymidine block and at 7.5 hrs after release (**d**). Auxin −, without Auxin; Auxin +, 0.5 µM of Auxin added. Movies are shown in Supplementary Movies 13–18. In (**b**,**c**,**e**), M-phase length for each observed cell is plotted. Bi, 100 nM of AZD1152; Cdc7i, 2 µM of PHA-767491; Cdc7ix, 2 µM of XL413; Noc, 30 ng/ml of Nocodazole; Pacli, 10 nM of paclitaxel. In (**b**), 10 µM PHA-767491 was also examined.

Aurora B is one of the important components of spindle assembly checkpoint (SAC). We next examined whether Cdc7 is involved in SAC. HeLa cells expressing GFP-HH2B was released into S phase for 8 hrs from thymidine-induced arrest. Then, drugs were added and M-phase progression was monitored by time-laps microscopy (Fig. 6a). Addition of a Cdc7 inhibitor (PHA-767491) increased M-phase duration in a dose-dependent manner, similar to the Aurora B inhibitor, AZD1152 (Fig. 6b, Supplementary Fig. S6a), consistent with a role of Cdc7 in M-phase. Addition of nocodazole or paclitaxel increased the length of M-phase, presumably by inducing SAC. In agreement with previous reports^39,40^, an Aurora B inhibitor (AZD1152) reduced the M-phase duration in the presence of nocodazole (from 450 min to 300 min). More significant effect of the Aurora B inhibitor was observed in the presence of paclitaxel (from 500 min to less than 50 min; Fig. 6c, Supplementary Movies 5, 8, 9 and 12).

Addition of a Cdc7 inhibitor (PHA-767491 or XL413) in the presence of nocodazole reduced M-phase length compared to that observed with nocodazole alone (from 450 min to 350 min). This is similar to the effect observed with the Aurora B inhibitor (AZD1152) in the presence of nocodazole (Fig. 6c, Supplementary Movies 5–8). Strikingly, the Cdc7 inhibitors, like the Aurora B inhibitor, dramatically reduced the paclitaxel-induced extension of M-phase (from 500 min to 100 min; Fig. 6c and Supplementary Fig. S6b, Supplementary Movies 9–12). These effects of Cdc7 inhibition were observed equally with two different Cdc7 inhibitors (PHA-767491 and XL413). AID-tagged mClover-Cdc7 cells were also treated with nocodazole or paclitaxel in the presence or absence of Auxin at 7.5 hrs after release from thymidine induced G1/S block. Extended M-phase induced by paclitaxel was significantly shortened in the presence of Auxin (Fig. 6d,e, Supplementary Movies 13 and 14**)**. However, there was a little effect of reduced Cdc7 level on M-phase length in nocodazole-treated AID-tagged Cdc7 cell line (Fig. 6d,e, Supplementary Movies 15 and 16). These results strongly suggest that Cdc7 is important also for SAC through modulating Aurora B activity.

## Discussion

### Cdc7 stimulates kinase activity of Aurora B *in vitro* by inducing phosphorylation of T236

Cdc7 is an evolutionally conserved kinase that plays an essential role in initiation of DNA replication by phosphorylating Mcm and Claspin^1,4–6,41–43^. Cdc7 is known to regulate other chromosomal events as well. Those include meiotic recombination, trans-lesion DNA synthesis and other DNA repair, meiotic cell division, histone modification, and recombination-dependent repair^17,44^. Thus, Cdc7 targets multiple proteins to modulate various biological functions. It is expressed also during G2-M of the vegetative growth, but its functions in this phase of cell cycle have been unknown.

Aurora B plays crucial roles in progression of M-phase. The functions of Aurora B are implicated in various M-phase events including spindle checkpoint, error correction, Plk1 activation, correct cytokinesis and others^28,45^. Aurora B is activated by various factors. Among them, INCENP plays a major role in activating Aurora B. The C-terminal region of INCENP (IN-box domain; aa803-918) is sufficient to bind and activate Aurora B^22,46^. Inhibition of Aurora B kinase prevents HH3 phosphorylation in prophase but not in metaphase, and results in delayed mitosis exit.

In the current study, we show that Cdc7 phosphorylates Aurora B and stimulates its phosphorylation activity *in vitro* and in cells. Mass spectrometry analyses of phosphorylated products *in vitro* suggest that T236 is one of the phosphorylation sites on Aurora B detected after phosphorylation by Cdc7. T236 of Aurora B is phosphorylated also *in vivo*, as indicated by the comprehensive phosphoprotein analysis^47^. The KD mutant (D200N) of Aurora B can be phosphorylated by Cdc7, albeit to a lower extent compared to the wild-type Aurora B (lanes 16 and 18 of Fig. 2e). The phosphorylation of KD by Cdc7 is significantly reduced by the TA mutation that inactivate the T236 phosphorylation (lane 4 of Fig. 2e), consistent with the idea that T236 may be directly phosphorylated by Cdc7. However, AT or DT derivative of KD carrying the intact T236 is also not very efficiently phosphorylated by Cdc7. Considering the fact that all the T232/T236 mutants exhibit compromised kinase activity (Fig. 2b, see below), it is likely that kinase activity of Auroa B is required for T236 to be efficiently phosphorylated. The KD-TT mutant is phosphorylated by Cdc7 to significant extent (lane 16 of Fig. 2e), but the T232 mutations reduced the level of phosphorylation (lanes 2, 8 and 12 of Fig. 2e). Therefore, the autophosphorylation at T232 may be required for efficient phosphorylation of T236 by Cdc7. It has been known that some phosphorylation events by Cdc7 depend on prior phosphorylation of the substrate by other kinases^41,48–50^. It should be noted that the KD mutant used in this study (D200N) still retains a low level of autophospohrylation activity (lane 6 of Fig. 2b), thus explaining KD-TT is phosphorylated by Cdc7 to a significant extent (lane 16 of Fig. 2e). (All the T232-T236 mutants in the D200N background show no autophosphorylation activity. The substitution of T232 with D or E apparently does not mimic the phosphorylation state in this case.) In conclusion, T236 may be a Cdc7 phosphorylation site, which is stimulated by prior autophosphorylation at T232. However, at present, we cannot completely exclude the possibility that T236 is an autophosphorylation site that stimulates Cdc7-mediated phosphorylation of Aurora B somewhere else. Generation of phospho-specific antibody that selectively recognizes phosphorylated T236 as well as detailed *in vitro* analyses of phosphorylation of Aurora B polypeptides by Cdc7 would be needed to obtain a definite answer to this issue.

### T232/T236 are required for Aurora B kinase activity

We have introduced a series of mutations at the T232/T236, and examined their phosphorylation activity. The results indicate that both T232 and T236 are essential for the full kinase activity of Aurora B. The replacement of these threonines with glutamic acid or aspartic acid did not result in a phosphomimic form, but in a kinase inactive form. This is not unusual. Indeed, we previously reported that replacement of threonine 376, a putative activating phosphorylation site, of budding yeast Cdc7 kinase with glutamic acid resulted in an attenuated kinase, not in an active kinase^51^.

INCENP is a co-activator of Aurora B. T232 and/ or T236 mutations did not affect the interaction with INCENP. Thus, T232/ T236 are required for the intrinsic kinase activity of Aurora B. Previous studies on the Aurora B-INCENP crystal structure in a complex with a small molecule inhibitor^22,52,53^ indicate that T232 and T236 are present in an activation T-loop of Aurora B. The phosphorylation of T236, which may be stimulated by prior autophosphorylation of T232, would induce conformational change of the Aurora B/INCENP complex that may activate its intrinsic kinase activity and potentially improve its substrate recognition.

### Cdc7 is required for full kinase activity of Aurora B in cells and for timely M-phase progression

We showed also that Cdc7 inhibition decreased the phosphorylation levels of two different substrates of Aurora B (Histone H3 Ser28 and CENP-A Ser7^54,55^) in M-phase cells, providing evidence for *in vivo* role of Cdc7 kinase for activation of Aurora B (Figs. 3 and 4). Furthermore, Cdc7 inhibition in early M-phase retarded M-phase progression (Fig. 5). On the other hand, error corrections of microtubule attachment to kinetochore were not significantly affected by Cdc7 inhibition in this experiment (Supplementary Fig. S5b).

### Cdc7 regulates spindle assembly checkpoint through Aurora B

Aurora B plays important roles also in SAC. Time laps images of M-phase progression in the presence of paclitaxel show prolonged M-phase and an Aurora B inhibitor reduced the M-phase duration (Fig. 6), as described previously^39,40^. We showed that inhibition of Cdc7 by small molecule inhibitors or Cdc7 depletion in Auxin-degron cell lines significantly reduced M-phase length of the cells arrested by paclitaxel, in much the same way as Aurora B inhibition did (Fig. 6).

The effect of Cdc7 inhibitors was more striking in Paclitaxel-treated cells than in nocodazole-treated cells (from 500 min to 100 min and from 450 min to 350 min, respectively; Fig. 6c). Degron-induced Cdc7 depletion also reduced M-phase duration in Paclitaxel-treated cells, but had no effect in nocodazole-treated cells (Fig. 6e).

This is consistent with the previous report on the effect of an Aurora B inhibitor on taxol or nocodazole treated cells. In the presence of an Aurora B inhibitor, taxol-arrested cells entered anaphase in less than one hr, whereas nococazole-treated cells stayed arrested for 3–5 hrs. These results are consistent with the idea that Cdc7 contributes to SAC through Aurora B activation. As previously shown, Aurora B inhibition in the presence of paclitaxel caused cytokinesis defect^39,40^. On the other hand, Cdc7 inhibition did not affect cytokinesis under the same condition (Fig. 6c). It was also recently reported that INCENP, as well as the interaction between Borearin and microtubule, are important for the SAC maintenance in taxol-treated human cells^56,57^. The requirement of Cdc7 for maintenance of taxol-induced SAC is likely through Aurora B, but at present, the possibility that Cdc7 is required for SAC maintenance in a manner independent of Aurora B cannot be excluded.

### Roles of Cdc7 kinases in M-phase progression

Aurora B localizes mainly on chromatin during early M-phase (Supplementary Fig. S7). Cdc7, present in nuclei during interphase^58,59^, localizes mainly in cytosol and partially on chromatin during M-phase (Supplementary Fig. S7), as indicated by the time laps analyses of fluorescence-tagged Cdc7 protein or by immunostaining^60^.

Cdc7 and Aurora B are partially colocalized but are present at distinct locations mostly during early M-phase (Supplementary Fig. S7).

We failed to show convincingly the binding between Aurora B and Cdc7 in coimmunoprecipitation experiments. This is probably because Cdc7 may phosphorylate Aurora B only during short time-span of the M-phase, and also because the association may be only transient. Previous studies indicate that Drf1/ASKL1, the second activation subunit of human Cdc7 the expression of which increases at late S to G2, is localized mainly in cytosol during M-phase^34^. Knockdown of Drf1/ASKL1 retarded M-phase progression likely through aberrant nuclear division and/or the failure of cytokinesis^34^, suggesting a possibility that the Cdc7-ASKL1 complex may regulate M-phase. In this study, we showed M-phase delay occurred rapidly after addition of a Cdc7 inhibitor or Cdc7 depletion in AID-derived cells in release from RO-3306-induced G2 phase (Fig. 5). Thus, Aurora B kinase may be activated during early M-phase by Cdc7-Drf1/ASKL1 for efficient M-phase progression.

Using a U2OS cell line stably expressing Kusabira-Orange-fused Aurora B, we showed that the Aurora B localization during M-phase is not affected by depletion of Cdc7 (data not shown). Cdc7 would be required for timely and full activation of Aurora B, probably by directly phosphorylating a threonine 236. Plk1, another M-phase kinase, is activated by phosphorylation at T210 present in its T-loop^61,62^. Aurora B is required for the T210 phosphorylation of Plk1 at centromeres in early mitosis^63,64^. Failure to activate Plk1 by Aurora B in prometaphase leads to defects in chromosome alignment and segregation^64,65^. Phosphorylation of Plk1 by Aurora B is required also for cytokinesis^66^. Thus, although Cdc7 does not directly affect the kinase activity of Plk1 and vice versa *in vitro* (Fig. 1b and Supplementary Fig. S1), Cdc7 depletion may affect the Plk1 kinase indirectly through Aurora B. In fact, the Plk1 pT210 signal on chromatin is reduced in Cdc7-depleted cells (data not shown).

Chk1 kinase, an S-phase checkpoint kinase, was reported to phosphorylates S331 of Aurora B during M-phase to increase Aurora B activity^67^. We show here that Aurora B is a target of another S-phase kinase, Cdc7, revealing a new layer of regulation of this important M-phase kinase. It should be noted that the Aurora B-INCENP complex is active without Cdc7 and Cdc7 augments its kinase activity. The effect of Cdc7 depletion on M-phase progression/ SAC can be noted but not as strong as that of Aurora or Plk1 kinase. Thus, Cdc7 probably fine-tunes M-phase progression and SAC by increasing Aurora B kinase activity.

## Materials and Methods

### Cells

U2OS (ATCC) and its derivative (*31–8*) in which Cdc7 shRNA can be inducibly expressed by addition of doxycycline were cultured in McCoy’s 5A medium containing 10% FCS. HeLa (ATCC) and its derivative (expressing EGFP-α-Tubulin, EGFP-CENP-A and H2B-mCherry; EGFP-CENP-A; EGFP-HH2B) were cultured in DMEM medium containing 10% FCS as described previously^4^. HCT116 and its derivative (HCT116-AID cMyc-Cdc7, AID mCl-Cdc7, HCT116-323) were cultured in McCoy’s 5A medium containing 10% FCS. HCT116-323 was constructed by introducing a 12 bp deletion in the promoter region of the Cdc7 gene by CRISPR-Cas9 (to be described elsewhere).

### Construction of AID-tagged Cdc7 derivative cell lines of HCT116 cells

Gene targeting using CRISPR/Cas9 was performed in HCT116 cells stably expressing *Oryza sativa* (Os)TIR1 as described previously^68,69^. The guide RNA sequence targeting Cdc7 locus was AAGATATGAGCTTGTGATAA(TGG) [protospacer adjacent motif (PAM) sequence is indicated in parentheses]. After selection with 0.4 mg/ml G418 and 0.1 mg/ml Hygromycin B Gold (Nacalai tesque Inc., Kyoto, Japan), isolated clones were screened by genomic PCR and immunoblotting as described previously^69^.

### Antibodies

Anti-Aurora B (ab2254), anti-INCENP (ab36453) and anti-HA (16B12; ab130275) antibodies were obtained from Abcam. Anti-Plk pT210 (sc-135706) and normal rabbit IgG were obtained from Santa Cruz Biotechnology. Anti-Cdc7 antibody was obtained from MBL. Anti-Aurora B pT232 antibody (Poly6361) obtained from BioLegend was used only for immunostaining. Anti-Histone H3 pS10 rabbit antibody was from Upstate. Anti-Histone H3 pS28 antibody was prepared in house in rat^70^. Anti-CENP-A pSer7 antibody (2187) was from Cell Signaling Technology. Antibodies against Tubulin, Aurora-A and FLAG (M2) were obtained from Sigma. Alexa 488 conjugated anti-rabbit IgG, Alexa 546 conjugated anti-rabbit IgG, Alexa 488 conjugated anti-rat IgG, and Alexa 647 conjugated anti-rabbit IgG, obtained from Invitrogen, were used for immune-staining or FACS staining.

### Chemicals

A Cdc7/Cdk9 inhibitor, PHA-767491, was used at 10 µM or 2 µM. Another Cdc7 inhibitor, XL413 (BioVision, BMS-863233), was used at 2 µM. Aurora B inhibitor (AZD1152, Sigma) was used at 100 nM. Nocodazole was used at 30, 50 or 100 ng/ml. Paclitaxel was used at 10 nM. RO-3306 (CDK1 inhibitor; MedChemexpress) was used at 10 µM. Auxin was used at 0.5 µM. Doxycycline was used at 1 µg/ml.

### Purified proteins

Human Aurora B (full-length wild-type, kinase dead [D200 to N] and other mutant forms) and the C-terminal domain (IN-box; amino acids 803–918)^71^ of INCENP were inserted at *Bam*HI site of ver.3–4 mammalian expression vector^72^ for generation of His- and Flag-double-tagged proteins (N-terminus and C-terminus, respectively). The INCENP segment was inserted also at *Bam*HI site in the ver.3–5 vector^72^ for generation of His- and HA-double-tagged protein. The plasmid DNAs were transfected into 293 T cells as described^72^. The cells were collected, extracted with CSK buffer (10 mM Pipes-KOH [pH 6.8], 100 mM potassium glutamate, 1 mM MgCl_2_, 1 mM EGTA, 300 mM sucrose, 1 mM dithiothreitol, 1 mM Na_3_VO_4_, 50 mM NaF, protease inhibitors [from Roche], 0.1 mM ATP and 0.1%Triton X-100) containing 300 mM NaCl, 0.5 mM PMSF, and 10 U/ml Benzonase (Amersham). The cell extracts were applied onto anti-Flag M2 affinity beads (Sigma, A2220). The column was washed three times with the wash buffer (50 mM sodium phosphate buffer [pH7.5], 300 mM NaCl, 10% Glycerol, 0.005% Triton X100, 0.2 mM PMSF and protease inhibitors [from Roche]). The purified protein was concentrated by using Microcon10. Human Aurora B/INCENP, Plk1 and Cdc7-ASK, expressed and purified from insect cells, were also purchased from Carna. Histone H3 was purchased from Roche.

We also generated the recombinant baculovirus encoding GST-tagged rat Aurora B (wild-type or kinase-dead) or His-tagged IN-box (amino acids 786–876)^71^ of mouse INCENP, using a combination of the GATEWAY^TM^ vector conversion system and the Bac-to-Bac baculovirus expression system (Invitrogen). GST-Aurora B (wild-type or kinase-dead) and INCENP-IN-Box were co-expressed in Sf9 insect cells through co-infection of the above baculoviruses. The complex of the two proteins (referred to as rat Aurora B-INCENP complex in Fig. 1f) was purified through affinity chromatography with glutathione-sepharose 4B (GE Healthcare, Little Chalfont Buckinghamshire, UK). The rat Aurora B was purified from Sf9 cells infected with the baculovirus encoding GST-tagged Aurora B alone (used in Figs. 1a and 3d).

### Kinase assays

Purified kinases and a substrate, as indicated in each figure legend, were incubated in kinase buffer containing ATP for 30 min at 30 °C as described previously^41,73^. For IP kinase assays, beads were equilibrated in kinase buffer (40 mM Hepes KOH [pH7.6], 0.5 mM EDTA, 0.5 mM EGTA, 1 mM β-glycerophosphate, 1 mM NaF, and 2 mM DTT) and were then incubated at 30 °C for 30 min in kinase buffer supplemented with ATP, magnesium acetate and Histone H3 or Histone H3.1 as a substrate. In non-radioactive kinase assays, the concentration of ATP was 0.5 mM, while it was 10 µM in assays with [γ-^32^P]ATP (unless stated otherwise in figures). For analyses on SDS-PAGE, reactions were stopped by addition of one-forth volume of 5x SDS sample buffer, heated at 96 °C for one min and applied onto SDS-PAGE.

### Mass spectrometry analysis

*In vitro* kinase assays were performed as described above. The proteins were separated on 4–20% gradient gel and stained by silver. Stained Aurora B proteins were extracted from gel, digested by Trypsin and phospho-threonines or serines were analyzed. The results are presented in Supplementary spreadsheet.

### Immunostaining

For immunostaining, cells were cultured on a glass-bottomed dish, fixed with 4% PFA (1% formaldehyde for CENP-A pS7 staining) for 10 min at room temperature and permeabilized with 0.1% Triton-X100 in PBS for 5 min, followed by wash with PBS. The fixed cells were stained by a primary antibody for overnight at 4 °C and then incubated with a secondary antibody conjugated with Alexa 488, 555 or 546. All antibodies were diluted with dilution buffer (2% BSA, 10% glycerol, 0.2% Tween20). Fixed and stained cells were observed by LSM confocal microscopy 710 or 780 (Carl Zeiss) and Z slice images were obtained. The Alexa 488 (green), GFP or Alexa555 (red) signals on chromatin (Hoechst33342) were analyzed by Imaris. The total signal intensity on chromatin from Z sliced images was analyzed. For analyses of CENP-A pS7 signal (red) on centromere (GFP-CENP-A, green), Imaris spot analysis was used.

### FACS analyses

For FACS analysis, cells were fixed in 70% ethanol-PBS, stained with a primary antibody, and then incubated with an Alexa-conjugated secondary antibody. Cells were further incubated with 20 µg/ml propidiumiodide and RNaseA in PBS, followed by analyses using a flow cytometer (FACS CantoII, BD).

### Live cell imaging

HeLa cells expressing EGFP-α-Tubulin, EGFP-CENP-A and H2B-mCherry were grown in glass bottom chambers (Thermo). Before imaging, the medium was changed to pre-warmed Leibovitz’s L-15 medium (Life Technologies) supplemented with 20% fetal bovine serum and 20 mM HEPES(pH 7.0). Recordings were made in a temperature-controlled incubator at 37 °C. In Fig. 5e–g, Z-series of five sections in 3-µm increments were captured every 2 min (Supplementary Movies 1 and 2). Image stacks were projected. All time-lapse images were collected with an Olympus IX-71 inverted microscope (Olympus) controlled by Delta Vision softWoRx (Applied Precision) using a ×20 0.75 NA UPlan SApochromat objective lens (Olympus) (Fig. 5e–g).

HeLa cells expressing EGFP-Histone H2B cells or AID-mClover-Cdc7 HCT116 cells were grown in glass bottom dish. LCV110 (Olympus) time-lapse microscopy was used to capture the images (Fig. 6)^74^.

## Supplementary information

Supplementary Information

Supplementary Movie 1

Supplementary Movie 2

Supplementary Movie 3

Supplementary Movie 4

Supplementary Movie 5

Supplementary Movie 6

Supplementary Movie 7

Supplementary Movie 8

Supplementary Movie 9

Supplementary Movie 10

Supplementary Movie 11

Supplementary Movie 12

Supplementary Movie 13

Supplementary Movie 14

Supplementary Movie 15

Supplementary Movie 16

Supplementary Movie 17

Supplementary Movie 18

Supplementary spreadsheet
