# Supplementary material for: Cdc7 kinase stimulates Aurora B kinase in M-phase
Source: Sci Rep. 2019 Dec 9;9:18622. doi: 10.1038/s41598-019-54738-2 (PMC6901529; doi:10.1038/s41598-019-54738-2)
Supplement: Supplementary file 1 — Supplementary Information [file 41598_2019_54738_MOESM1_ESM.pdf]

Title: Cdc7 kinase stimulates Aurora B kinase in M-phase

Authors: Sayuri Ito[1], Hidemasa Goto[2], Kinue Kuniyasu[3], Mayumi Shindo[4], Masayuki Yamada[5,6], Kozo Tanaka[3], Gaik-Theng Toh[1], Masaaki Sawa[7], Masaki Inagaki[8], Jiri Bartek[6,9,10], Hisao Masai[1,11]

1. Department of Genome Medicine, Tokyo Metropolitan Institute of Medical Science, Tokyo 156-8506, Japan.
2. Department of Neural Regeneration and Cell Communication, Mie University Graduate School of Medicine, Tsu, Mie 514-8507, Japan.
3. Department of Molecular Oncology, Institute of Development, Aging and Cancer, Tohoku University, Sendai 980-8575, Japan.
4. Laboratory of Protein Analyses , Tokyo Metropolitan Institute of Medical Science, Tokyo 156-8506, Japan
5. Medical Education Center, Graduate School of Medicine, Kyoto University, Kyoto, Japan.
6. Institute of Molecular and Translational Medicine, Faculty of Medicine and Dentistry, Palacky University, 77900, Olomouc, Czech Republic.
7. Carna Biosciences, Inc., Kobe, Japan.
8. Department of Physiology, Mie University Graduate School of Medicine, Tsu, Mie 514-8507, Japan.
9. Danish Cancer Society Research Center, Copenhagen, Denmark.
10. Division of Genome Biology, Department of Medical Biochemistry and Biophysics, Karolinska Institute, Stockholm, Sweden.
11. To whom correspondence should be addressed

Correspondence should be addressed to
Hisao Masai
Department of Genome Medicine, Tokyo Metropolitan Institute of Medical Science, 4-6-1 Kamiktazawa, Setagaya-ku, Tokyo 156-8506, JAPAN
E-mail: masai-hs@igakuken.or.jp
Telephone: +81-3-5316-3220
Fax: +81-3-5316-3145

Ito *et al*. Supplementary Figure S1

a

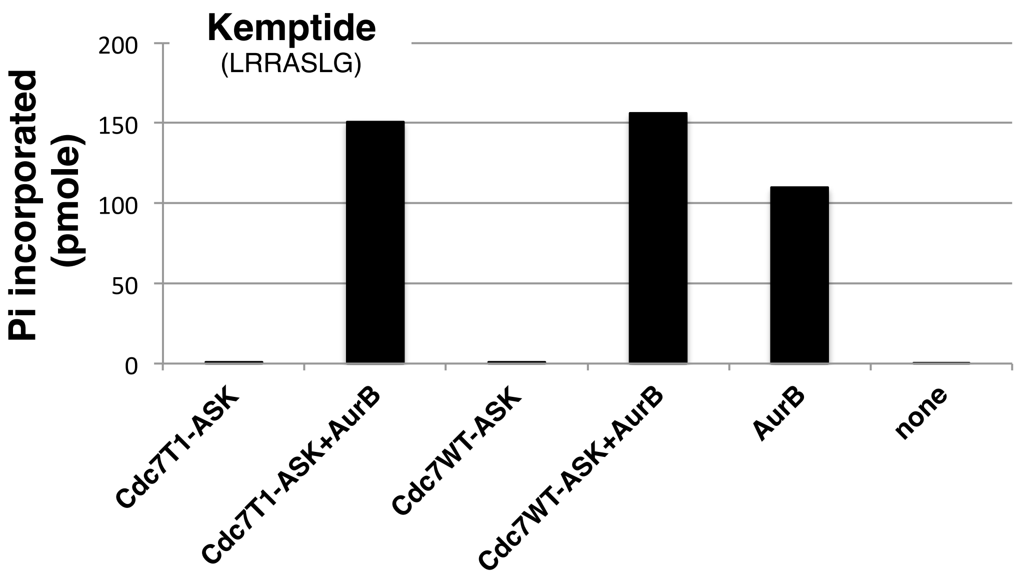

b

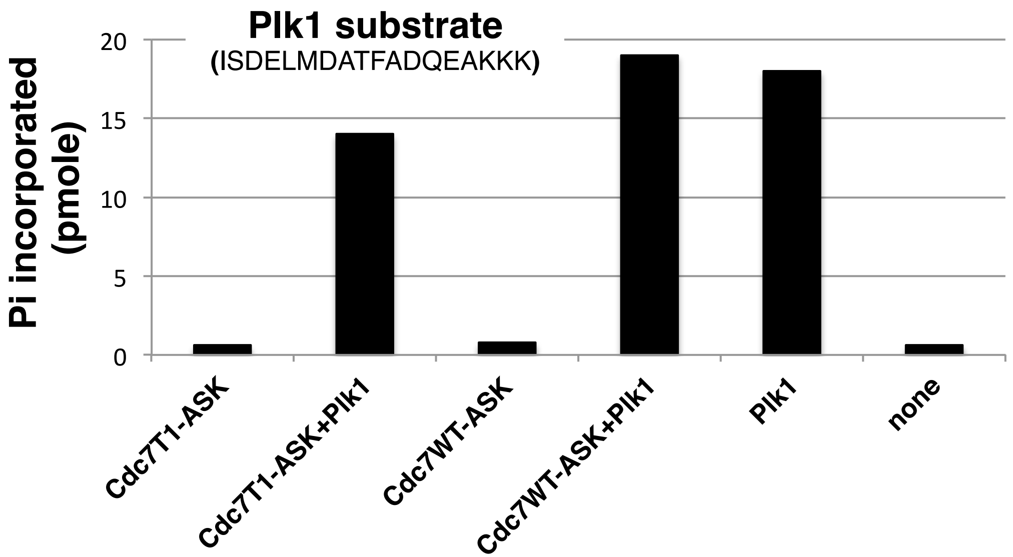

Supplementary Figure S1 *In vitro* kinase assays with Cdc7-ASK, Aurora B and Plk1 kinases (related to Fig. 1).
In vitro kinase assays were conducted with the combinations of the kinases shown and a radioactive ATP, using Kemptide (**a**) or Plk1 substrate (**b**) peptide (200 pmole each) as a substrate. The amount of kinases are as follows; Cdc7T1-ASK[1] (expressed in *E. coli*), 100 ng (1.5 pmole); Cdc7-ASK[2] (expressed in insect cells), 20 ng; Aurora B (from Carna Inc.), 20 ng; Plk1 (from Carna Inc.), 20 ng. The incorporation was measured by spotting an aliquot of the reaction mix on P81 filter paper, followed by wash in 75 mM phosphoric acid, and measuring the radioactivity remaining on the filters.

Ito *et al.* Supplementary Figure S2

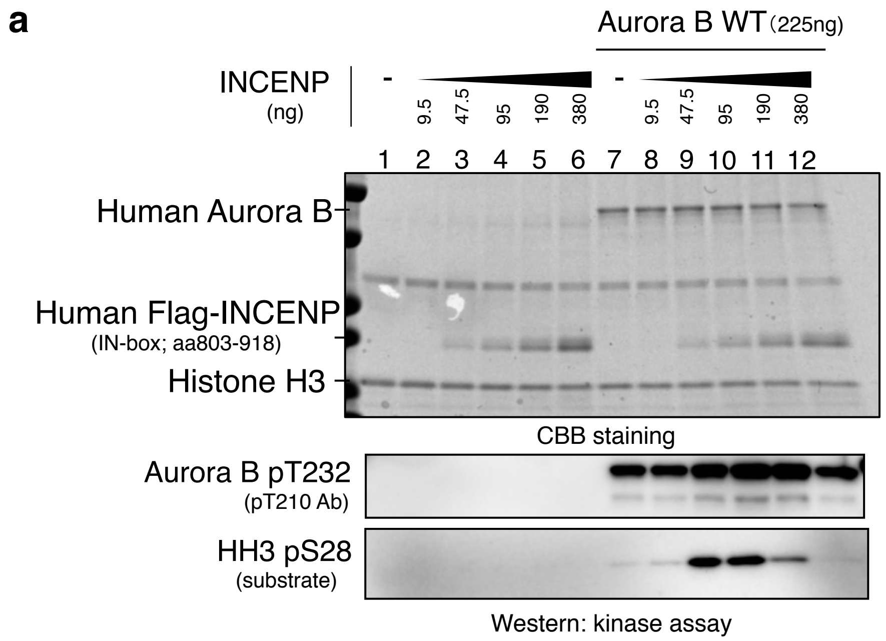

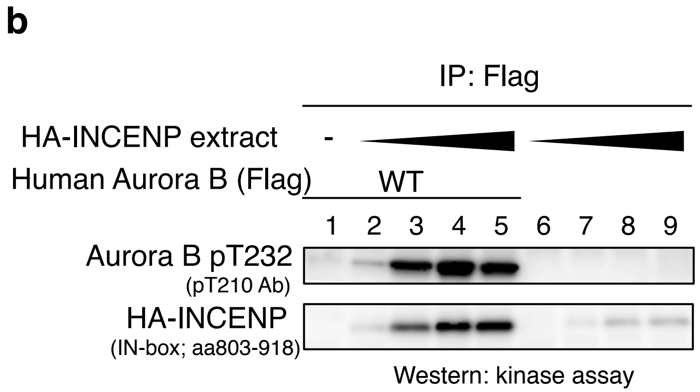

Supplementary Figure S2 INCENP binds and stimulates Aurora B (related to Fig. 2).
(**a**) *In vitro* kinase assays were performed with Aurora B and Histone H3 in the presence of increasing amounts of Flag-tagged INCENP (IN-box polypeptide; amino acids 803-918) (lanes 1 and 7, no INCENP; lanes 2 and 8, 9.5 ng; lanes 3 and 9, 47.5 ng; lanes 4 and 10, 95 ng; lanes 5 and 11, 190 ng; lanes 6 and 12, 380 ng). The products were run on SDS-PAGE and were analyzed by Western and CBB staining. Anti-HH3 pS28 and anti-pT210 antibodies were used for western to detect phosphorylation. (**b**) Aurora B-Flag bound to anti-Flag beads were mixed with the 293T cell extract expressing HA-INCENP (IN-box polypeptide). Beads were washed three times and were used for kinase assays with HH3 as a substrate (phosphorylation detected by anti-pT210 and anti-HH3 pS28 antibodies) and pulled-down INCENP was analyzed by Western.

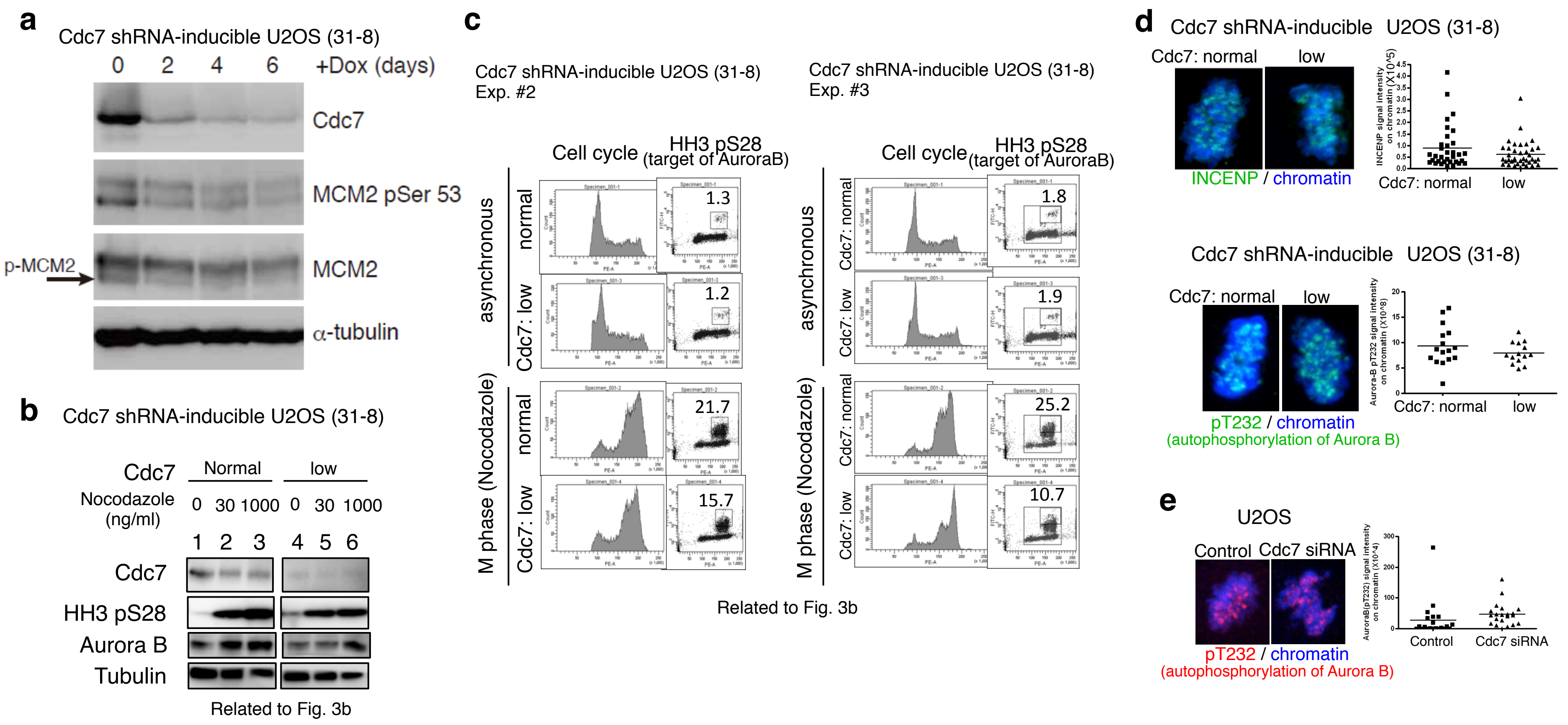

Supplementary Figure S3 Cdc7 stimulates Aurora B kinase activity in M phase cells (related to Fig. 3).
(a) *31-8* cells, in which Cdc7 expression can be repressed by inducible shRNA, were incubated with 1 µg/ml doxycycline for days indicated. Cdc7 and phosphorylation of MCM2 Ser53 (a target of Cdc7 kinase) in the whole cell extracts were examined by Western. The arrow indicates the phosphorylated form of Mcm2 which is known to be generated by Cdc7 kinase. **(b)** *31-8* cells incubated with (low Cdc7) or without (normal Cdc7) doxycycline were either treated with nocodazole (at indicated concentrations) for 23 hrs before harvest. The whole cell extracts were analyzed by Western. **(c)** Different independent sets of experiments for **Fig. 3b** are shown. The numbers indicate the populations (%) of HH3 pS28-positive cells. **(d)** *31-8* cells were fixed and stained. Upper: green, INCENP; blue, chromatin (Hoechst33342). Lower, green, pT232; blue, chromatin. **(e)** U2OS cells treated with Cdc7 siRNA or control siRNA for 48 hrs were stained with anti-pT232 (red) and Hoechst33342 (blue, chromatin). Cdc7-D siRNA (GCAGUCAAAGACUGUGGAUTT)[3] was used. The sense strand of Cdc7-D siRNA was used for control transfection. **(f)** AID-tagged mClover-Cdc7 HCT116 cells were treated with Auxin for 1.5 hrs, and cell extracts were prepared with CSK buffer containing 300mM NaCl. The supernatant fractions were analyzed by Western. These extracts were used for IP-kinase assays in **Fig. 3g**.

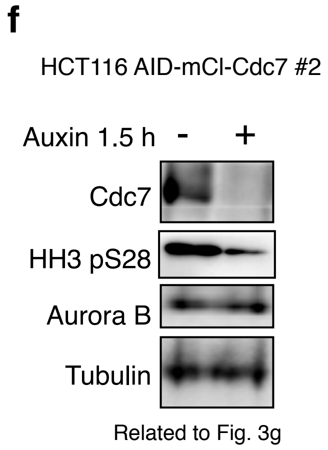

Ito *et al.* Supplementary Figure S3

Ito *et al*. Supplementary Figure S4

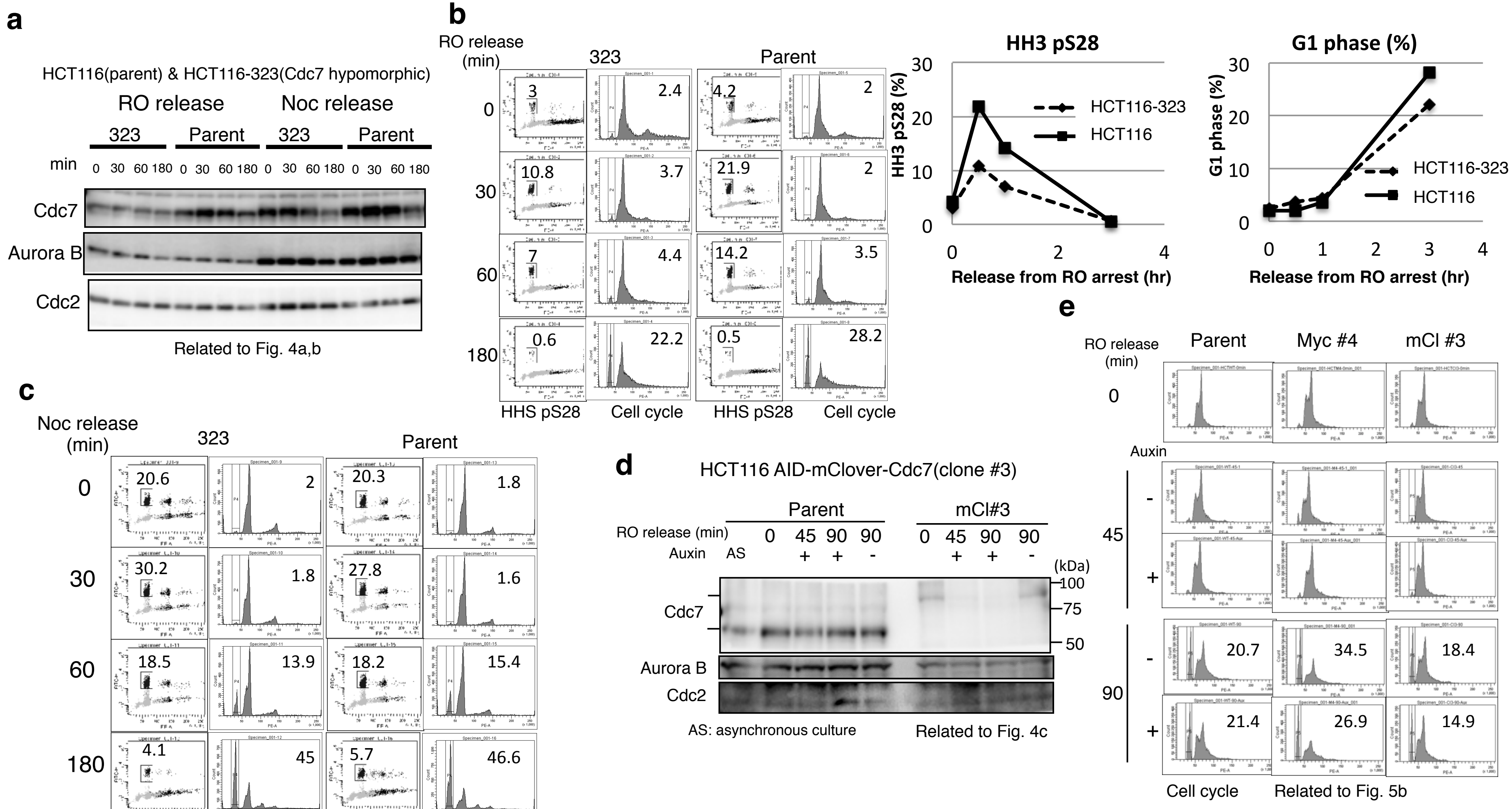

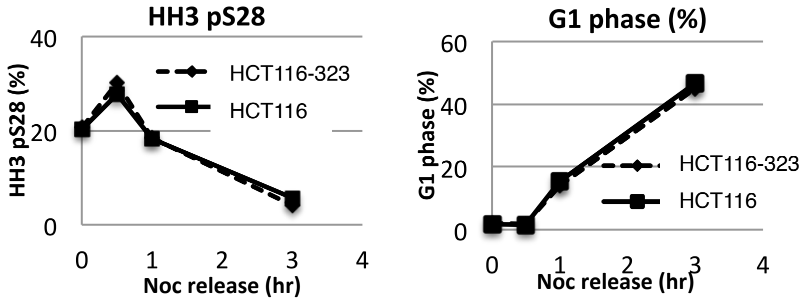

Supplementary Figure S4 Western blot and FACS analyses of cells released from RO-3306 or Nocodazole arrest (related to Fig. 4 and 5)

(**a, b, c**) HCT116 and HCT116-323 (Cdc7 hypomorphic cell line) were released from RO-3306 (**a,b**) or nocodazole(**c**)-mediated arrest. (**a**) Western analysis of the whole cell extracts. (**b,c**) The samples from (**a**) were analyzed by FACS. **(b)** is a duplicate experiment of **Fig. 4a**. (**d**) AID-mClover Cdc7 #3 or HCT116 parent cells were treated with Auxin for the indicated time after release from RO-3306. The whole cell extracts were analyzed by Western using antibodies indicated. This is a part of the samples used for **Fig. 4c**. (**e**) The original FACS data of **Fig. 5b**. The numbers in the panels of FACS data indicate the populations (%) of HH3 pS28-positive or G1 phase cells.

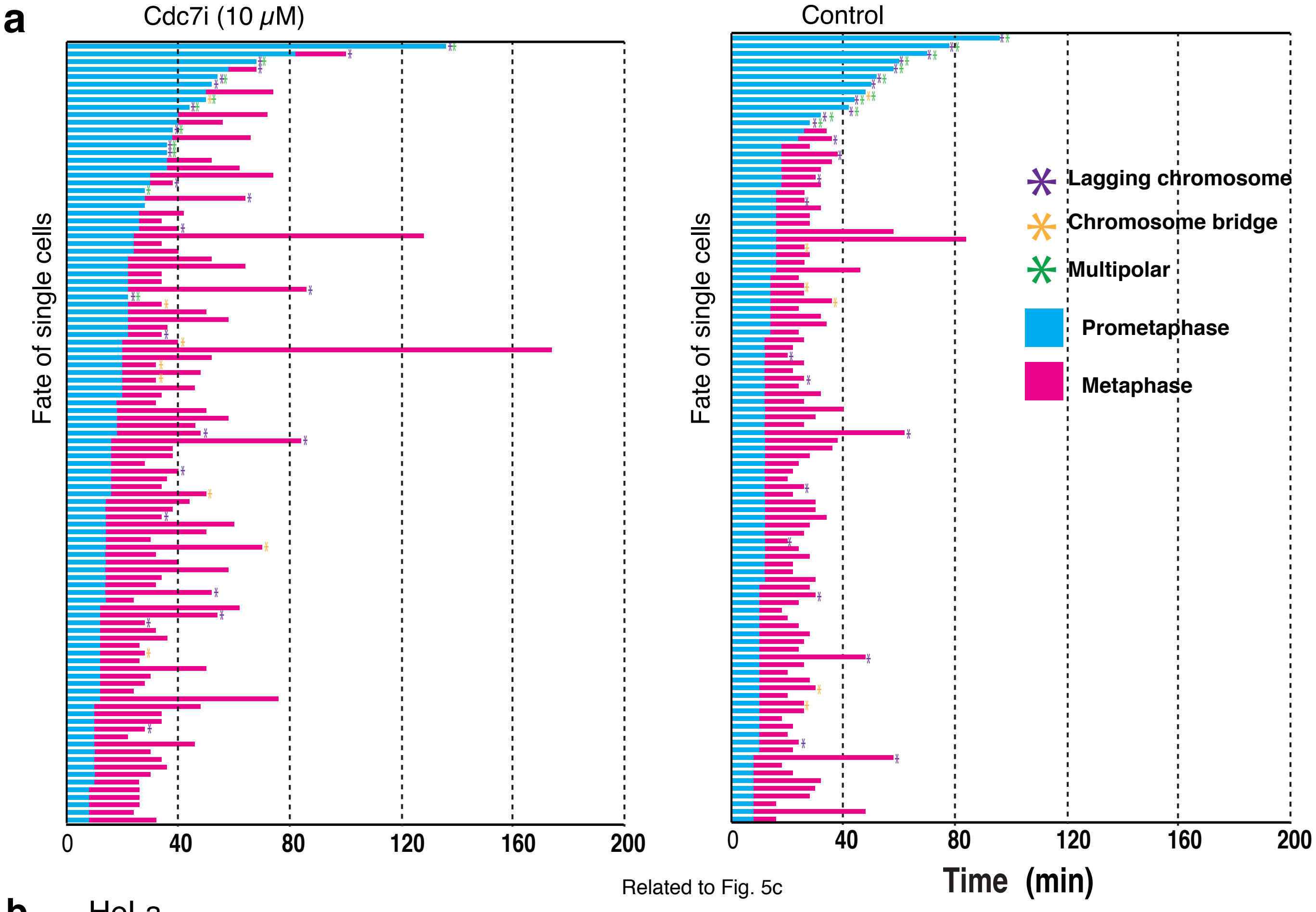

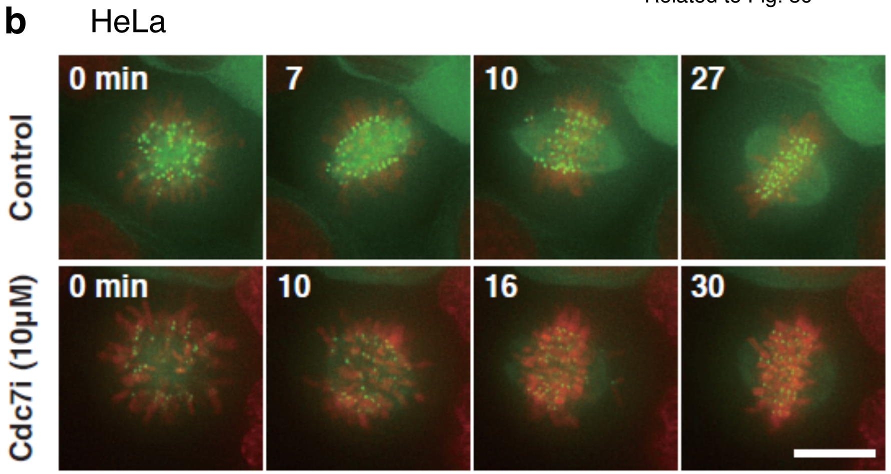

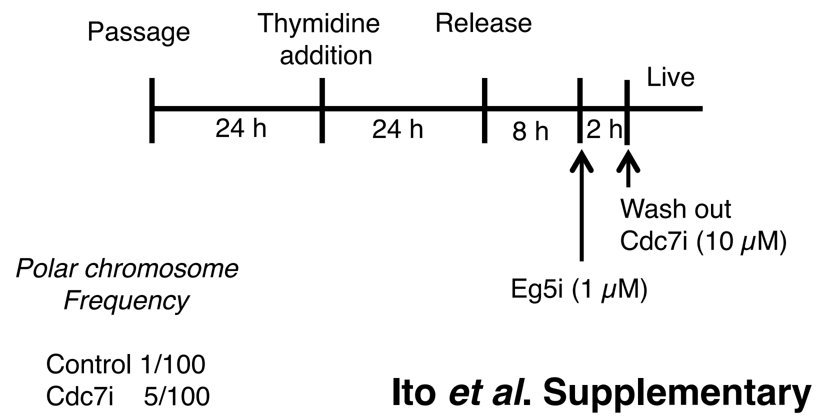

Supplementary Figure S5
Effect of Cdc7 inhibition on M-phase chromosomes
(**a**) The lengths of prometaphase, metaphase are shown for control and Cdc7-depleted cells (103 single cells for each). Cells with abnormal chromosomes are also indicated. This is an original data of **Fig. 5g**. (**b**) At 8 hrs after release from thymidine block, cells were treated with 1 µM Eg5 inhibitor for 2 hrs. Then cells were washed and non-treated or treated with 10 µM Cdc7 inhibitor (PHA-767491). Live cell images were monitored with an Olympus IX-71 inverted microscope (Olympus) controlled by Delta Vision softWoRx (Applied Precision) using a ×60 1.42 N Plan Apochromat objective lens (Olympus) and polar chromosomes were counted[4] (**Supplementary Movies 3, 4**). Representative cells are shown for control and Cdc7i-treatedcells. Numbers indicate time (min) after washout of Eg5. Scale bar, 10 µm.

Ito *et al.* Supplementary Figure S5

Ito *et al*. Supplementary Figure S6

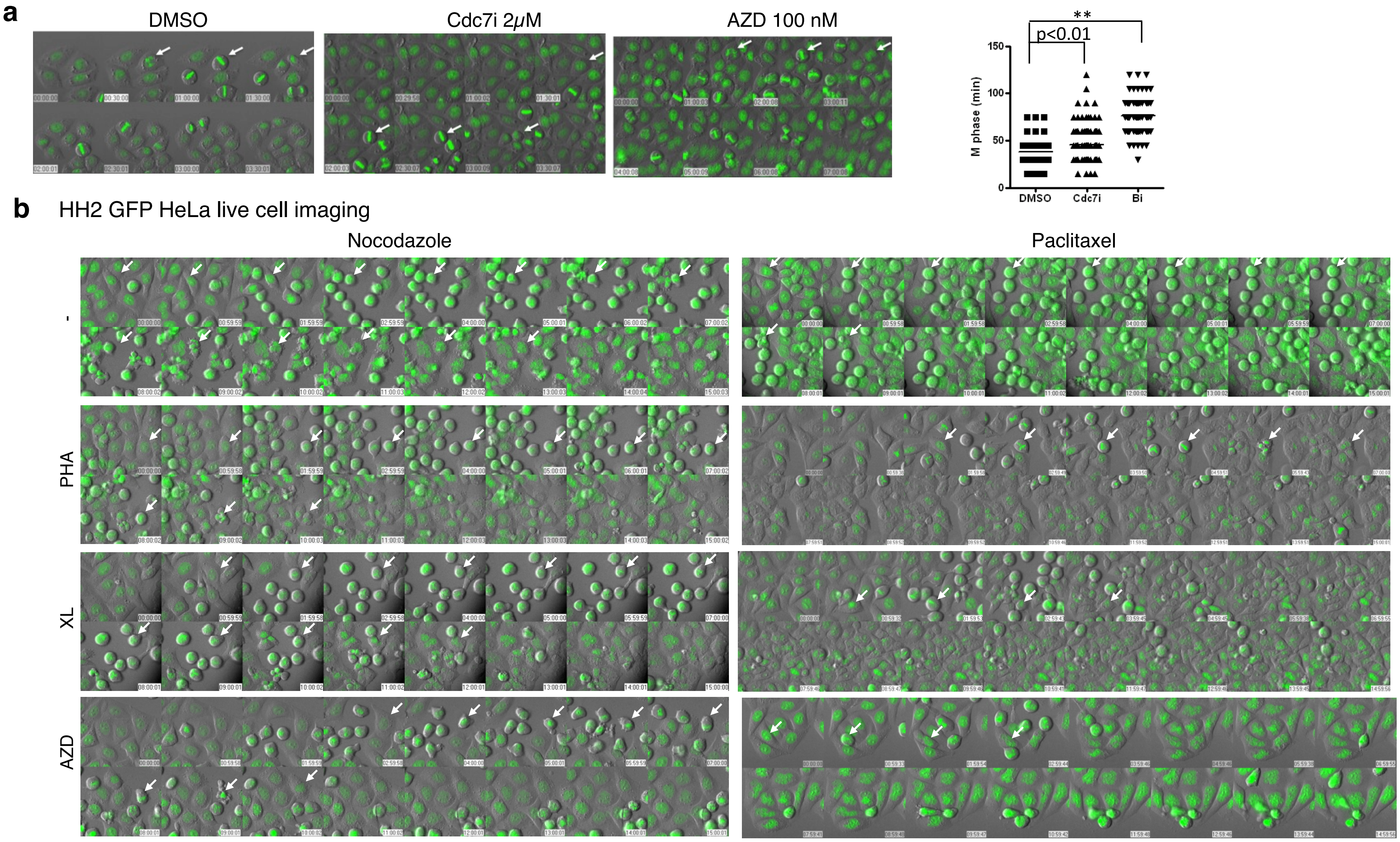

Supplementary Figure S6 Effect of Cdc7 inhibition with Nocodazole or paclitaxel (related to Fig. 6).
At 7.5-8 hrs after the release from thymidine block, HeLa cells expressing HH2B-GFP were treated with various drugs as indicated, and live cell images were monitored. M phase duration of each cell was counted from the movie. (**a**) DMSO, Cdc7 inhibitor (Cdc7i, PHA767491, 2 μM) or Aurora B inhibitor (Bi, AZD1152, 2 μM) were added. This is another set of the experiments identical to the one shown in **Fig. 6b**. ** $p<0.0001$ (**b**) Representative images from the movies (**Supplementary Movies5-12**) showing the chromosomes of nocodazole or paclitaxel-treated cells in the presence of various inhibitors. White arrows indicate the cells in mitotic phase. M phase duration was measured for each cell and presented in **Fig. 6c**.

Ito *et al*. Supplementary Figure S7

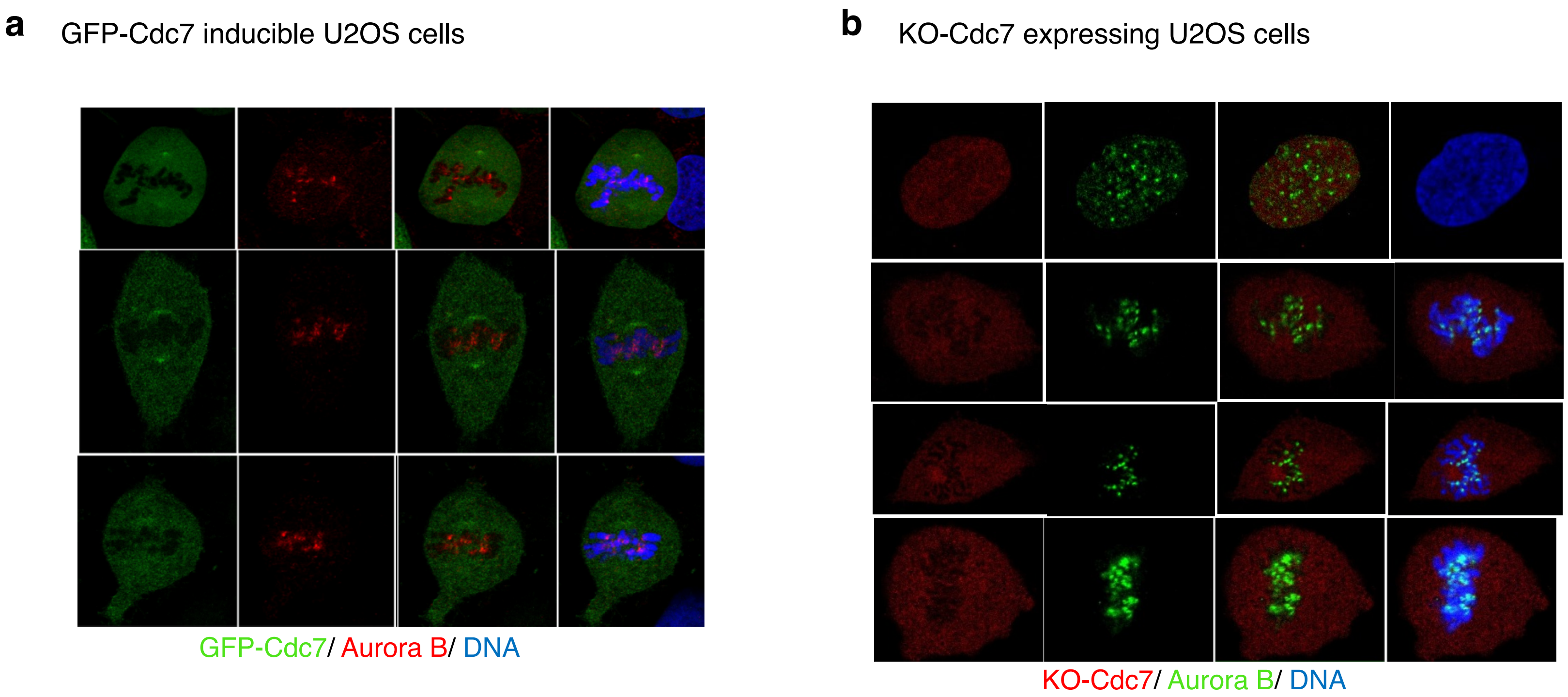

Supplementary Figure S7 Cellular localization of Aurora B and Cdc7 during early M-phase.
(**a**) U2OS cells in which GFP-Cdc7 can be induced by doxycycline were fixed and stained with Aurora B antibody (Alexa 546). GFP-Cdc7, green; Aurora B, red; Hoechst33342 (DNA), blue. (**b**) U2OS cells that stably express KO-Cdc7 were fixed and stained with Aurora B antibody (Alexa 488). KO-Cdc7, red; Aurora B, green; Hoechst33342 (DNA), blue. In both (**a**) and (**b**), early M-phase cells were selected and observed by Olympus FV3000 microscopy. Aurora B signals are very similar to those previously reported[5].

Ito *et al*. Supplementary Figure S8

Fig.3d original

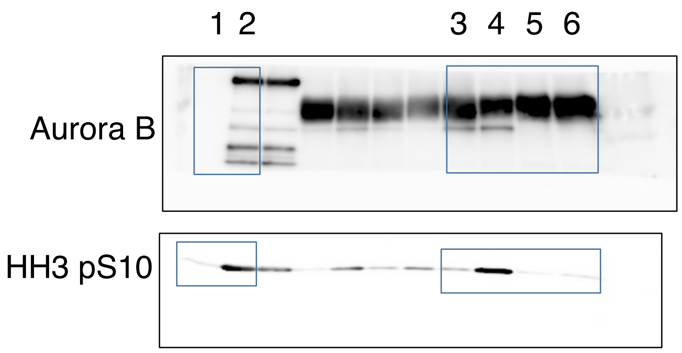

Fig.3e original

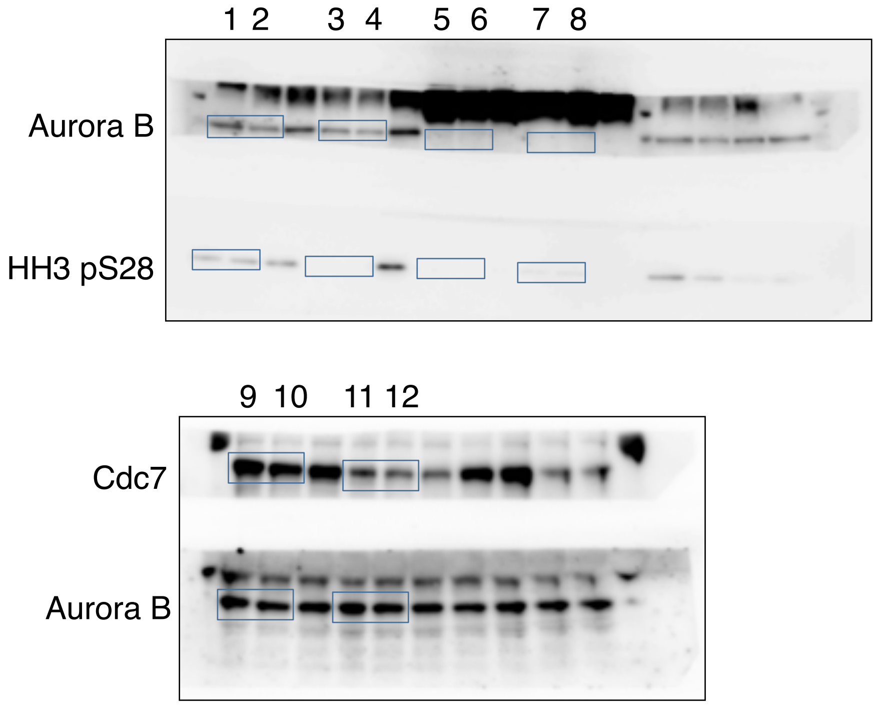

Fig.2f long exposure image

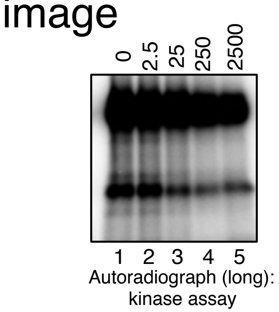

Fig.3g long exposure image

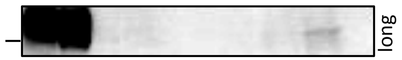

Supplementary Figure S8 Full-length blots for Figure 3d and 3e and long exposure images for Fig. 2f and Fig. 3g. The locations of the cropped images shown in **Fig. 3d and Fig. 3e** are indicated on the original full-length blots.

Ito *et al*. Supplementary Figure S9

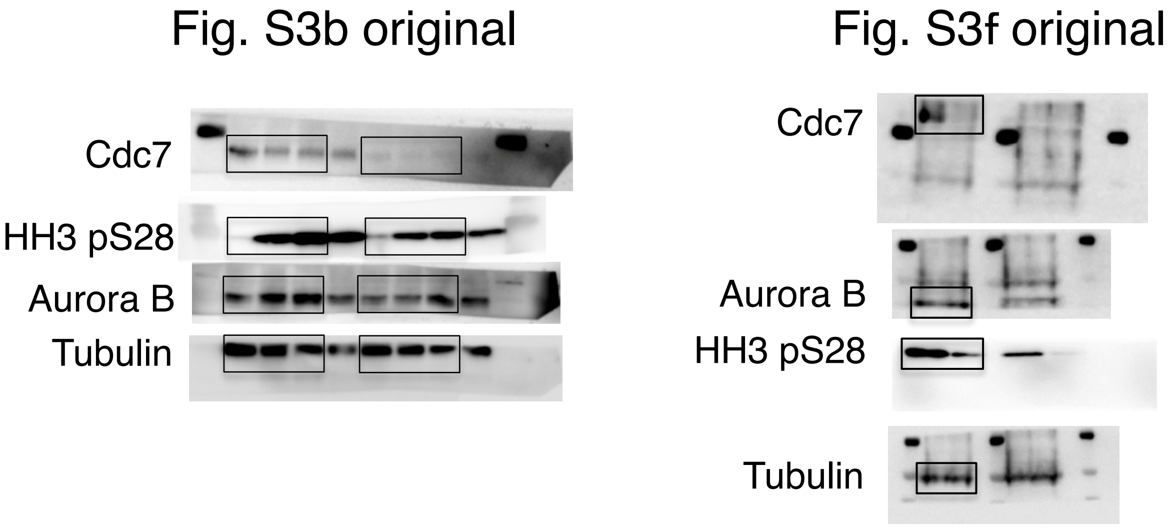

Supplementary Figure S9 Uncropped blots for Figure S3b and S3f. The locations of the cropped images shown in Fig. S3b and Fig. S3f are indicated on the uncropped blots.

Supplementary Movie 1 Mitotic progression of HeLa cells expressing EGFP-α-tubulin, EGFP-CENP-A, and H2B-mCherry. Movie speed is 7 frames per second.
Supplementary Movie 2 Mitotic progression of HeLa cells expressing EGFP-α-tubulin, EGFP-CENP-A, and H2B-mCherry in the presence of a Cdc7 inhibitor, PHA-767491 (10 μM). Movie speed is 7 frames per second.
Supplementary Movie 3 Mitotic progression of HeLa cells expressing EGFP-α-tubulin, EGFP-CENP-A, and H2B-mCherry released from treatment with Eg5 inhibitor III (1 μM). Movie speed is 7 frames per second.
Supplementary Movie 4 Mitotic progression of HeLa cells expressing EGFP-α-tubulin, EGFP-CENP-A, and H2B-mCherry released from treatment with Eg5 inhibitor III (1 μM) in the presence of a Cdc7 inhibitor, PHA-767491 (10 μM). Movie speed is 7 frames per second.
In the Supplementary Movies 1-4: Green, EGFP; Red, mCherry

Supplementary Movie 5-12: Mitotic progression of HH2B expressiong HeLa cells
Supplementary Movie 5: 30 ng/ml Nocodazole
Supplementary Movie 6: 30 ng/ml Nocodazole with Cdc7 inhibitor (PHA-767491) 2uM
Supplementary Movie 7: 30 ng/ml Nocodazole with Cdc7 inhibitor (XL413) 2uM
Supplementary Movie 8: 30 ng/ml Nocodazole with Aurora B inhibitor (AZD1152) 100nM
Supplementary Movie 9: 10nM Paclitaxel
Supplementary Movie 10: 10nM Paclitaxel with Cdc7 inhibitor (PHA-767491) 2uM
Supplementary Movie 11: 10nM Paclitaxel with Cdc7 inhibitor (XL413) 2uM
Supplementary Movie 12: 10nM Paclitaxel with Aurora B inhibitor (AZD1152) 100nM
Supplementary Movie 13-18: Mitotic progression of AID-mClover-Cdc7 #2 cells
Supplementary Movie 13: 10nM Paclitaxel
Supplementary Movie 14: 10nM Paclitaxel with Auxin
Supplementary Movie 15: 30 ng/ml Nocodazole
Supplementary Movie 16: 30 ng/ml Nocodazole with Auxin
Supplementary Movie 17: new medium
Supplementary Movie 18: new medium with Auxin

Supplementary spreadsheet
Aurora B proteins incubated in the kinase assay conditions with the following combinations of proteins were analyzed by mass spectrometry.
1. Rat Aurora B (wild-type) with Histone H3
2. Rat Aurora B (wild-type) and Cdc7-ASK complex (from Carna) with Histone H3
3. Rat Aurora B (kinase-dead) with Histone H3
4. Rat Aurora B (kinase-dead) and Cdc7-ASK complex (from Carna) with Histone H3
5. Human Aurora B-INCNEP complex (from Carna) and Plk1 (from Carna)
6. Human Aurora B-INCNEP complex (from Carna)
7. Human Aurora B-INCNEP complex (from Carna), Plk1 (from Carna) and Cdc7-ASK complex (from Carna)
8. Human Aurora B-INCNEP complex (from Carna) and Cdc7-ASK complex (from Carna).

Human Aurora B pT232 and rat Aurora B pT231 are shown in blue, and human Aurora B pT236 and rat Aurora B pT235 are shown in red.

References

(1) Kitamura, R. *et al.* Molecular mechanism of activation of human Cdc7 kinase: bipartite interaction with Dbf4/activator of S-phase kinase (ASK) activation subunit stimulates ATP binding and substrate recognition. ***J Biol Chem*** **286**, 23031-23043, doi:10.1074/jbc.M111.243311 (2011).
(2) Masai, H. *et al.* Human Cdc7-related kinase complex. In vitro phosphorylation of MCM by concerted actions of Cdks and Cdc7 and that of a criticial threonine residue of Cdc7 by Cdks. ***J Biol Chem*** **275**, 29042-29052, doi:10.1074/jbc.M002713200 (2000).
(3) Ito, S. *et al.* Mechanism of cancer cell death induced by depletion of an essential replication regulator. ***PLoS One*** **7**, e36372, doi: 10.1371 /journal.pone.0036372. (2012)
(4) Kim, Y., Holland, A. J., Lan, W. & Cleveland, D. W. Aurora kinases and protein phosphatase 1 mediate chromosome congression through regulation of CENP-E. ***Cell*** **142**, 444-455, doi:10.1016/j.cell.2010.06.039 (2010).
(5) Murata-Hori, M. *et al.* Probing the dynamics and functions of aurora B kinase in living cells during mitosis and cytokinesis. ***Mol Biol Cell*** **13**, 1099-1108 (2002).
